# The association of common autoimmune diseases with autoimmune thyroiditis: a two-sample Mendelian randomization study

**DOI:** 10.3389/fendo.2024.1383221

**Published:** 2024-09-09

**Authors:** Kaiyuan Zhang, Ziyue Luo, Xinchang Wang

**Affiliations:** ^1^ Second Clinical Medical College, Zhejiang Chinese Medical University, Hangzhou, Zhejiang, China; ^2^ Department of Rheumatology, The Second Affiliated Hospital, Zhejiang Chinese Medical University, Hangzhou, Zhejiang, China

**Keywords:** Mendelian randomization, causality, genetic, autoimmune thyroiditis, autoimmune diseases

## Abstract

**Objective:**

Numerous observational and retrospective studies have demonstrated an association between Autoimmune Thyroiditis (AIT) and various systemic Autoimmune Diseases (AIDs). However, the causal relationship between them remains uncertain. This study aims to investigate the causal link between AIT and diverse types of AIDs utilizing the Mendelian Randomization (MR) method.

**Method:**

We assessed the causal relationship between AIT and eight prevalent AIDs. Summary statistics from genome-wide association studies (GWAS) were sourced from the FinnGen biobank and IEU Open GWAS database. Two-sample MR analyses were conducted, with the primary statistical approach being the Inverse Variance Weighting (IVW) method. This was complemented by a series of sensitivity analyses, and the robustness of the findings was evaluated through the estimation of heterogeneity and pleiotropy.

**Results:**

When AIT was considered as the outcome, MR evidence suggested an association between Rheumatoid arthritis (RA), Type 1 diabetes (T1D), and Systemic lupus erythematosus (SLE) with AIT. Utilizing the Inverse Variance Weighting (IVW) method, we observed an increased risk of AIT with exposure to RA (*P* = 0.024, OR=1.25; 95% CI = 1.03, 1.52), T1D (*P* < 0.001, OR=1.27 95% CI = 1.11,1.46), and SLE (*P* = 0.037, OR=1.14; 95% CI = 1.04,1.26). Conversely, no significant genetic causal relationship with AIT was found for Sjögren’s syndrome (SS), Ankylosing Spondylitis (AS), Multiple sclerosis (MS), Crohn’s disease (CD), and Ulcerative colitis (UC).

**Conclusion:**

This study identified RA, T1D, and SLE as triggering factors for AIT. The incidence rate of AIT in patients with RA, T1D, and SLE may be higher than that in the general population. Therefore, individuals with these three diseases should undergo regular monitoring of thyroid-related indicators.

## 1 Introduction

Autoimmune thyroiditis (AIT) is an autoimmune thyroid disease (AITD) characterized by the immune system targeting the thyroid gland, with Hashimoto’s thyroiditis (HT) being the most prevalent clinical manifestation (1). Epidemiological reports indicate that AIT is among the most common autoimmune diseases (AIDs) globally, affecting approximately 3-5% of individuals worldwide, and its incidence continues to rise (2).

The key features of AIT include the presence of thyroid-specific autoantibodies, extensive lymphocyte infiltration, necrosis of thyroid cells, and destruction of follicular structures. Excessively necrotic thyroid tissue can release signals that induce immune responses in AIT. Thyroid follicular epithelial cells (TFCs) serve as target cells for the AIT immune response. When stimulated by pathogen-associated molecular patterns (PAMPs) and danger-associated molecular patterns (DAMPs), these cells express Toll-like receptors, leading to the overactivation of immune-related pattern recognition receptors (PRRs). Self-reactive lymphoid cells, primarily CD4+ T cells, aggregate and infiltrate the thyroid gland, causing autoimmune damage (3). Currently, AIT is a major cause of hypothyroidism (4). Moreover, individuals with AIT are more prone to cardiovascular disease and malignant tumors (5, 6). Therefore, identifying potential pathological factors that may trigger AIT holds promise for introducing novel ideas and methods for its diagnosis and treatment.

Epidemiological studies have indicated that a significant proportion of patients with AIT are more susceptible to comorbidities with other systemic rheumatic and immune diseases, including Type 1 diabetes (T1D), Rheumatoid arthritis (RA), Systemic lupus erythematosus (SLE), Multiple sclerosis (MS), and Sjögren’s syndrome (SS) (7). Moreover, several studies have highlighted that patients with other AIDs are also prone to an elevated incidence of AIT. Specifically, the incidence rate of AITD in RA patients has shown an increase of 1-6 times, with RA patients experiencing a less favorable initial treatment outcome (8).. Additionally, a cross-sectional study revealed that the incidence of AITD in 17% - 30% of adult patients with T1D reached 4% - 18% (9). A recent retrospective study revealed that 15.7% of patients with SS exhibit autoimmune inflammation in the thyroid, while 5.6% of patients have latent autoimmune inflammation. AIT and SS share common pathological and physiological characteristics, characterized by lymphocyte infiltration, particularly CD4^+^ T lymphocytes and B cells (10). Another retrospective case-control study conducted in China assessed SLE patients with AITD compared to SLE patients without AITD, revealing that anti-dsDNA positivity, serositis, and low complement C3 were associated with SLE patients having AITD. Notably, serositis was identified as a risk factor for AITD (11). The incidence of AITD in SLE patients is significantly higher than that in the general population.

In a longitudinal study, the occurrence of new cases of thyroid dysfunction and autoimmune diseases was assessed in 179 women with MS. Compared to the control group, the MS group exhibited a higher prevalence of thyroid dysfunction, specifically hypothyroidism, and autoimmune diseases (12). It is evident that AIT is closely associated with the onset and progression of various AIDs, which suggests a potential shared genetic basis among these conditions. However, despite epidemiological findings indicating associations between AIT and other AIDs, whether these associations imply a causal relationship remains unclear. Increasing evidence suggests that AIDs may manifest distinct clinical features and prognoses when combined with AIT. Some studies propose that this phenomenon may arise from common susceptibility genes and environmental triggers. However, this fails to fully elucidate the interaction between AIT and other AIDs (13). Therefore, it is crucial to determine whether a more profound causal relationship exists between AIT and other AIDs.

Mendelian Randomization (MR) is an innovative epidemiological method that relies on extensive Genome-Wide Association Study (GWAS) datasets. It utilizes Single Nucleotide Polymorphisms (SNPs) as instrumental variables (IVs) to unveil causal relationships (14). Currently, an increasing number of studies have embraced MR analysis to investigate the interconnections among various diseases. In this study, GWAS data for AIT were acquired for analysis, and a two-sample MR method was employed to explore the genetic underpinnings of the causal relationship between AIT and common Autoimmune Diseases (AIDs), including RA, SS, SLE, MS, T1D, Ankylosing spondylitis (AS), Ulcerative colitis (UC), and Crohn’s disease (CD).

## 2 Materials and methods

### 2.1 Study design

**Figure 1:**
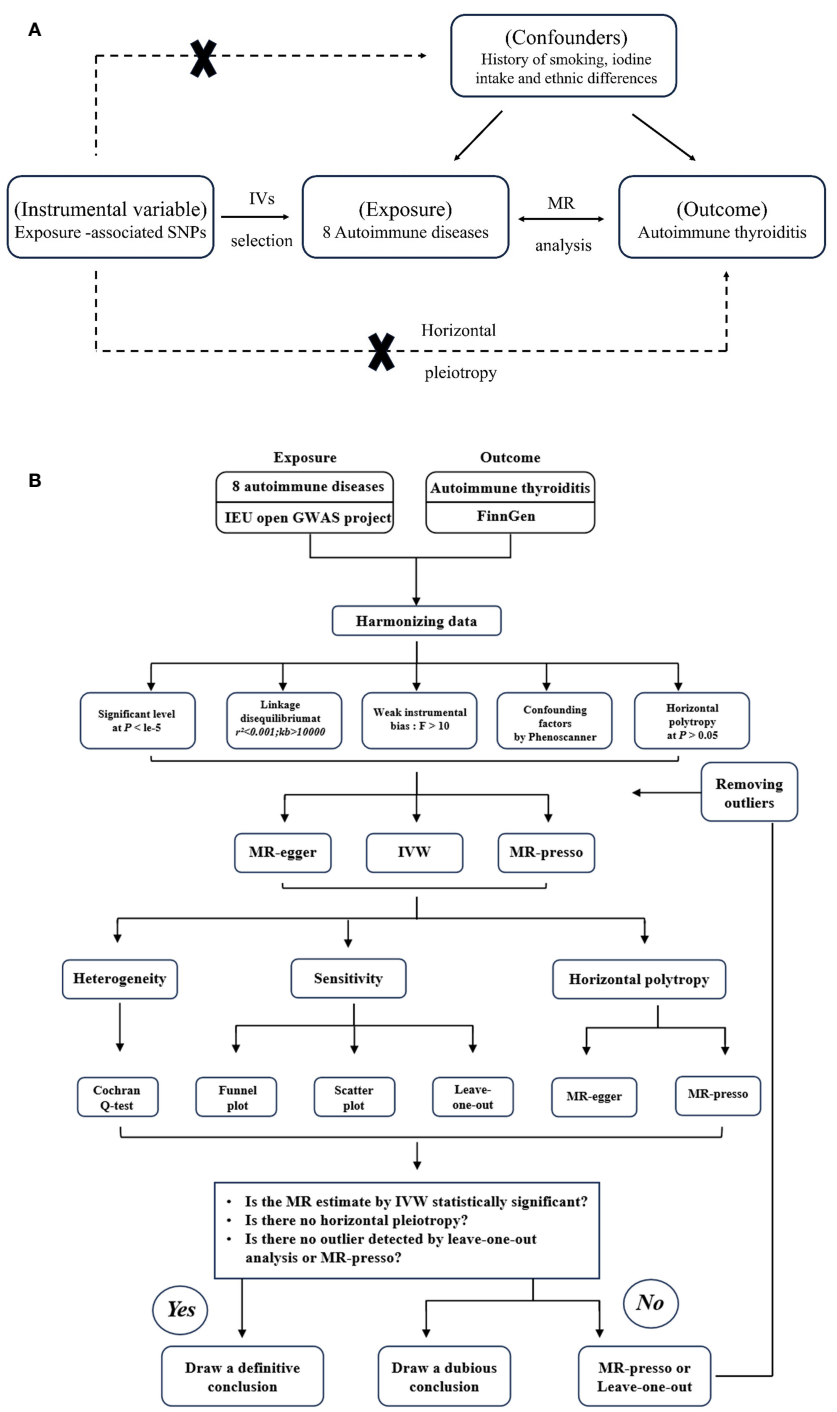
**(A)** The principles for mendelian randomization study are as follows: (1) genetic instrumental variables are associated with the exposure, (2) genetic instrumental variables are independent of confounding variables, (3) genetic instrumental variables only affect outcome through exposure. **(B)** The flowchart of the study.

We conducted two-sample MR analyses involving eight AIDs (AS, RA, SS, SLE, MS, T1D, UC, and CD) and AIT. The analysis process involved utilizing the eight autoimmune diseases as the exposure and AIT as the outcome for MR analysis. It is important to note that this study adheres strictly to the fundamental assumptions of MR analysis, which include the following: 1) establishing a robust relationship between IVs and exposure, 2) ensuring that IVs are independent of potential confounding factors, and 3) confirming that IVs only exert a direct impact on the results through exposure. The flowchart is depicted in **Figures 1A, B**.

### 2.2 Data source

The GWAS summary data of AS, RA, SS, SLE, MS, T1D, UC, and CD were sourced from the IEU Open GWAS database, accessible at https://gwas.mrcieu.ac.uk/. The GWAS summary data concerning AIT were obtained from the Finnish consortium, as found at https://www.finngen.fi/. It is crucial to underscore that all participants in AIDs and AIT cohorts share a common European ancestry. Furthermore, the data utilized in this study are entirely derived from publicly available databases. Hence, given the public accessibility of the data, no ethical declaration or informed consent form is required for this study. Comprehensive details concerning the implemented data are provided in **Supplementary Table 1**.

### 2.3 IVs selection

As commonly acknowledged, the meticulous selection of IVs forms the bedrock for ensuring the robustness of MR analysis results. Initially, IVs showed a strong correlation with exposure, and the criteria for establishing a significant correlation were defined as follows: *P*<1 x 10^-5^, F statistic>10. The calculation of the F-statistic uses the formula: F = R^2^(N-K-1)/K(1-R^2^). Subsequently, a thorough screening process was conducted to alleviate the impact of linkage disequilibrium (LD), retaining only SNPs displaying the least LD (LD r^2 < 0.001 and a clump distance > 10,000 kb) as IVs. Moreover, the chosen IVs must not exhibit a significant correlation with the outcomes, necessitating the removal of SNPs related to the outcomes based on the correlation threshold: *P*<1 x 10^-5^. To further mitigate confounding variables, the PhenoScanner database was utilized to alleviate the influence of extraneous factors (15). Common risk factors for AIT considered in this study included but were not limited to, smoking, iodine intake, ethnic differences, immunosuppressants, and endocrine disruptors (16). Ultimately, SNPs characterized by palindromic sequences and intermediate allele frequencies were systematically excluded from the analytical process.

### 2.4 Mendelian randomization analysis

The primary analysis method employed in this study is the Random Effects Inverse Variance Weighted (IVW) method (17). Additionally, MR Egger (18), weighted median (19), simple mode, and weighted mode methods (20) were employed as supplementary analyses. Heterogeneity was evaluated using Cochran’s Q test. In instances where heterogeneity was detected, a random effects IVW approach was adopted. The MR Egger regression test was utilized to evaluate horizontal pleiotropy. Intercepts represent pleiotropic effects across genetic variants (21). We also employed the MR polytropic residuals and outliers (MR-PRESSO) test to identify polytropic SNPs, corrected for horizontal pleiotropy by eliminating outliers. A distortion test was conducted to assess whether causal estimates before and after correcting for outliers were significantly different (22). Additionally, a “leave-one-out” analysis was performed to evaluate the stability of our findings.

### 2.5 Statistical analysis

The “TwoSampleMR” package was employed for two-sample MR analyses, and the “MRPRESSO” package was utilized for MR-PRESSO tests. All statistical analyses were conducted using R version 4.1.2. A significance level of *P* < 0.05 was set to infer genetic causation. Specifically, a *P*-value < 0.05 and an odds ratio (OR) > 1 indicated positive genetic causation, while an OR < 1 suggested a negative genetic causal association. Furthermore, a *P-* value > 0.05 signified the absence of heterogeneity and horizontal pleiotropy, aligning with the assumption of normal distribution.

## 3 Results

### 3.1 Selection of instrumental variables

#### 3.1.1 Rheumatoid arthritis

A total of 86 SNPs showed a strong correlation with RA as determined by the criterion of substantial correlation (*P* < 1 x 10^-5^ and F > 10). Further screening yielded 79 SNPs that showed a correlation with the AIT. Then proceeded to exclude one SNP (rs2476601) with pval.outcome < 1 x 10^-5^. Confounding SNPs were then excluded, resulting in the removal of one confounding SNP (rs71508903). Then proceeded to exclude 11 palindromic SNPs (palindromic results showed TRUE, rs231806, rs2647166, rs3093017, rs34536443, rs4410848, rs56074741, rs58107865, rs6930052, rs7089017, rs74625075 and rs78019882), and finally the remaining 66 SNPs were designated as IVs, as detailed in **Supplementary Table 2**.

#### 3.1.2 Ankylosing Spondylitis

A total of 42 SNPs showed a strong correlation with AS (*P* < 1 x 10^-5^ and F > 10). Further screening yielded 41 SNPs that showed a correlation with the AIT. No SNPs with pval.outcome < 1 x 10^-5^. No palindrome SNPs or confounder SNPs were found. Finally, the remaining 40 SNPs were designated as IVs, as detailed in **Supplementary Table 3**.

#### 3.1.3 Sjögren’s syndrome

A total of 17 SNPs showed a strong correlation with SS (*P* < 1 x 10^-5^ and F > 10). Further screening yielded 15 SNPs that showed a correlation with the AIT. Then proceeded to exclude one SNP (rs1811197) with pval.outcome < 1 x 10^-5^. There was no confounder SNPs. Then proceeded to exclude 1 palindromic SNPs (rs149449770), and finally the remaining 13 SNPs were designated as IVs, as detailed in **Supplementary Table 4**.

#### 3.1.4 Systemic lupus erythematosus

in the context of SLE, we obtained a team of 29 SNPs that exhibited strong associations. A set of 26 corresponding SNPs was extracted from the GWAS summary data of AIT. No SNPs with pval.outcome < 1 x 10^-5^. There was no confounder SNPs were excluded. Then proceeded to exclude 6 palindromic SNPs (palindromic results showed TRUE, rs115526225, rs371642845, rs4642071, rs539937225, rs72712464, and rs77063044). Finally, the remaining 20 SNPs were designated as IVs, as detailed in **Supplementary Table 5**.

#### 3.1.5 Multiple sclerosis

Subsequently, our investigation of MS generated 67 SNPs that exhibited strong statistical associations. Further screening yielded 65 SNPs that showed a correlation with the AIT. No SNPs with pval.outcome < 1 x 10^-5^ and no confounder SNPs were found. Then proceeded to exclude 7 palindromic SNPs (palindromic results showed TRUE, rs10896028, rs11060994, rs117861023, rs4525910, rs73720868, rs767626, and rs79638626), and finally the remaining 58 SNPs were designated as IVs, as detailed in **Supplementary Table 6**.

#### 3.1.6 Type 1 diabetes

We found a total of 64 SNPs in the context of T1D, which showed strong correlation with T1D. Further screening yielded 63 SNPs that showed a correlation with the AIT. Then proceeded to exclude one SNP (rs6679677) with pval.outcome < 1 x 10^-5^. Confounder SNPs were then excluded, and 6 confounded SNPs (rs1456988, rs1534422, rs2111485, rs3087243, rs3184504, and rs72928038) were screened out. No palindrome SNPs were found. Finally, the remaining 56 SNPs were designated as IVs, as detailed in **Supplementary Table 7**.

#### 3.1.7 Ulcerative colitis

A total of 81 SNPs showed a strong correlation with UC as determined by the criterion of substantial correlation (*P*< 1 x 10^-5^ and F > 10). Further screening yielded 71 SNPs that showed a correlation with the AIT. Then proceeded to exclude one SNP (rs2476601) with pval.outcome < 1 x 10^-5^. No confounder SNPs were found. Then proceeded to exclude 8 palindromic SNPs (palindromic results showed TRUE, rs12638810, rs151180560, rs181316459, rs297202, rs4065985, rs4817983, rs7936434, and rs889100), and finally the remaining 63 SNPs were designated as IVs, as detailed in **Supplementary Table 8**.

#### 3.1.8 Crohn’s disease

A total of 131 SNPs showed a strong correlation with CD as determined by the criterion of substantial correlation (*P*< 1 x 10^-5^ and F > 10). Further screening yielded 121 SNPs that showed a correlation with the AIT. Then proceeded to exclude one SNP (rs2476601) with pval.outcome < 1 x 10^-5^. No confounder SNPs were found. Then proceeded to exclude 17 palindromic SNPs (palindromic results showed TRUE, rs11564236, rs116475860, rs12194825, rs12692254, rs148844907, rs1541953, rs17643535, rs1775452, rs1887428, rs2581828, rs2675670, rs3933040, rs6588052, rs6762648, rs7714401, rs78487399, rs8178977, and rs9573018), and finally the remaining 102 SNPs were designated as IVs, as detailed in **Supplementary Table 9**.

**Figure 2:**
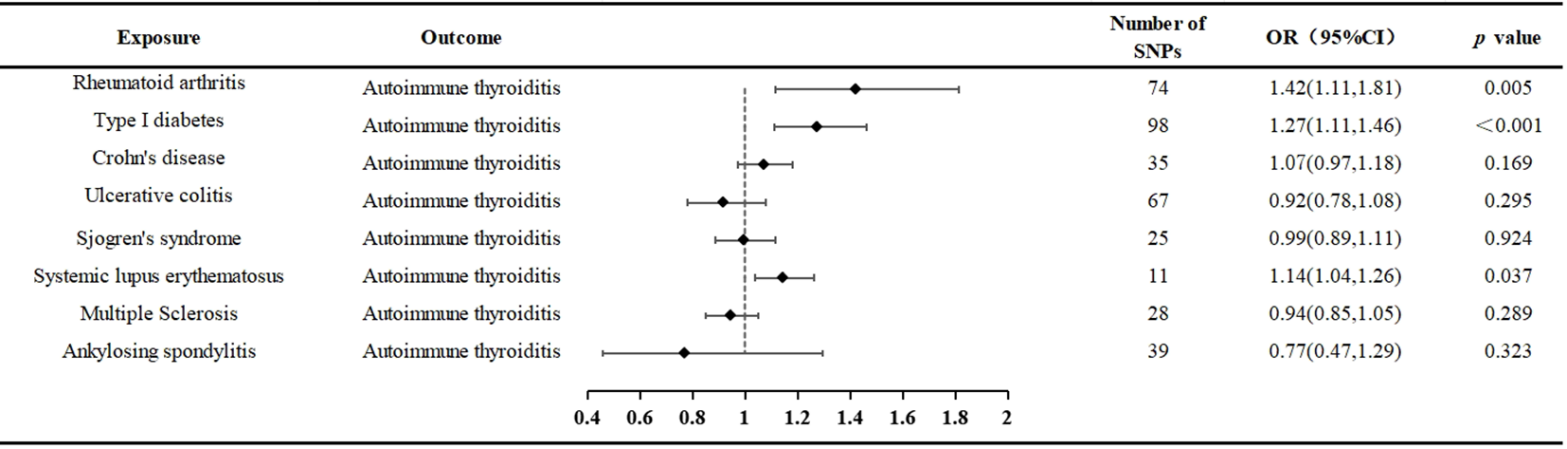
The selection process of instrumental variable. Firstly, A genome-wide significance threshold *P*< 1 × 10^−5^ was used, and Linkage disequilibrium (LD) test was performed using PLINK, and LD r ^2^ < 0.001 was adopted to ensure the independence of the selected genetic variants. Secondly, The F-statistic of each SNP was calculated, and SNPs with F < 10 were eliminated to avoid weak instrument bias. Thirdly, we loaded the GWAS Mendelian data package of the outcome indicator AIT in and obtained the SNPs related to the outcome. Fourthly, we excluded SNPs with pval.outcome < 1 x 10^-5^ from the outcome. Fifthly, we used PhenoScanner to further exclude confounding SNPs. Finally, we removed SNPs with palindromic results of TRUE.

The comprehensive program adopted for selecting instrumental variables visually depicts **Figure 2**.

### 3.2 Genetic causality between exposures (AS, RA, SS, SLE, MS, T1D, UC, and CD) and outcome (AIT)

#### 3.2.1 Rheumatoid arthritis

**Figure 3:**
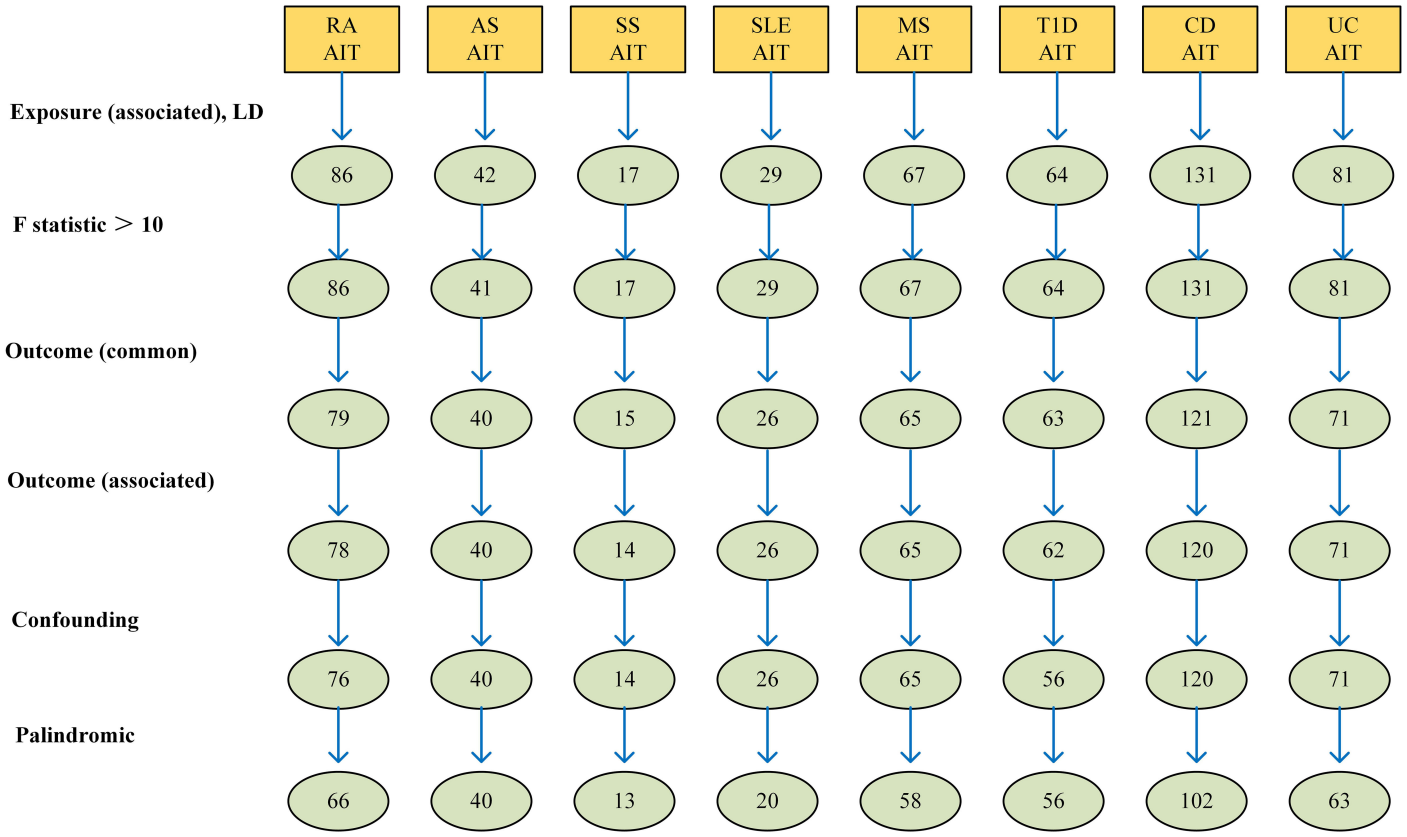
Forest plots of the causal effect of AIDs on AIT. Autoimmune thyroiditis (AIT); rheumatoid arthritis (RA); Type 1 diabetes(T1D); systemic lupus erythematosus (SLE), Sjögren’s syndrome (SS), Ankylosing Spondylitis (AS), Multiple sclerosis (MS), Crohn’s disease (CD); ulcerative colitis (UC).

**Figure 4:**
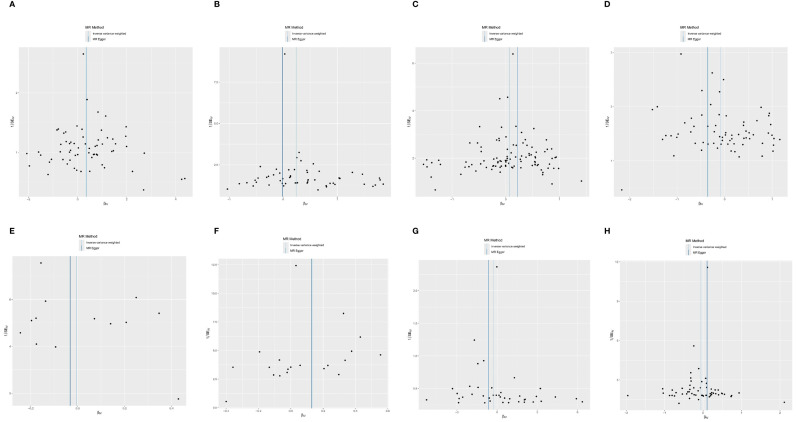
The scatter plot of MR analysis between exposures (RA, T1D, CD, UC, SS, SLE, AS, and MS) and outcome (AIT). **(A)** RA and AIT; **(B)** T1D and AIT; **(C)** CD and AIT; **(D)** UC and AIT; **(E)** SS and AIT; **(F)** SLE and AIT; **(G)** AS and AIT; **(H)** MS and AIT.

The outcomes of the random-effects IVW analysis revealed a positive genetic causal relationship between RA and AIT (*P* = 0.024, odds ratio [OR] = 1.25; 95% confidence interval [CI] = 1.03, 1.52) (**Figure 3**). Cochran’s Q statistic for the MR-IVW approach and Rucker’s Q statistic for the MR Egger method were used to assess heterogeneity; both showed no significant heterogeneity (*P* > 0.05). The MR Egger intercept test showed that horizontal pleiotropy was not present (*P* > 0.05). However, horizontal pleiotropy was detected using the MR-PRESSO test (*P* < 0.05) (**Supplementary Table 10**). The MR-PRESSO test identified two significant outliers (rs35139284 and rs9277398), further supporting this finding. After excluding the notable outliers, a second iteration of the MR analysis was conducted considering the detected horizontal pleiotropy. The re-evaluated IVW outcomes found a positive genetic causal connection between RA and AIT (*P* = 0.005, OR 95% CI = 1.42[1.11, 1.81]). Additional methods, including the weighted median method, simplified model method, and MR Egger method, showed a consistent direction of influence between RA and AIT (**Figure 4A**, **Supplementary Table 11**).

#### 3.2.2 Type 1 diabetes

The outcomes of the random-effects IVW analysis revealed a positive genetic causal relationship between T1D and AIT (*P* < 0.001, OR=1.27 95% CI = 1.11,1.46) (**Figure 3**). The absence of heterogeneity (*P* > 0.05) and the absence of horizontal pleiotropy, as determined through the intercept test of MR Egger (*P* > 0.05). MR-PRESSO test also indicated that the absence of horizontal pleiotropy (*P >*0.05) (**Supplementary Table 10**). No significant outliers are in MR-PRESSO test. Additional methods showed a consistent direction of influence between T1D and AIT (**Figure 4B**, **Supplementary Table 11**).

#### 3.2.3 Systemic lupus erythematosus

The results of the IVW analysis with random effects showed a favorable genetic causal connection between AIT and SLE (*P* = 0.037, OR=1.14; 95% CI = 1.04,1.26) (**Figure 3**). The lack of horizontal pleiotropy (*P* > 0.05) and the absence of heterogeneity (*P* > 0.05), as shown by the MR Egger intercept test. Additionally, the MR-PRESSO test revealed that there was no horizontal pleiotropy *(P* > 0.05) (**Supplementary Table 10**). The results of the four supplementary analyses were consistent with those of the IVW analysis (**Figure 4F**, **Supplementary Table 11**).

#### 3.2.4 Crohn’s disease and ulcerative colitis

The random-effects IVW analysis revealed that there exists no significant genetic causal relationship between UC (*P* = 0.295, Odds OR 95% CI = 0.92 [0.78, 1.08]) and CD (*P* = 0.169, OR=1.07; 95% CI = 0.97, 1.18) in relation to AIT (**Figure 3**). Four supplementary analyses consistently aligned with the outcomes of the random-effects IVW analysis (**Figures 4C, D**, **Supplementary Table 11**). The evaluation of genetic causality involving UC, or CD, in conjunction with AIT, exhibited uniformity in terms of the absence of heterogeneity (*P >*0.05), horizontal pleiotropy (*P >*0.05), and outliers (*P >*0.05) (**Supplementary Table 10**).

There was no significant genetic causal relationship between AIT and these AIDs. (**Figure 3**, **Supplementary Table 10**). The results of the four supplementary analyses were consistent with those of the random effects IVW analysis (**Figures 4C–H**).

#### 3.2.5 Sjögren’s syndrome and ankylosing spondylitis

SS (*P* = 0.924, Odds OR=0.99; 95% CI = 0.89, 1.11) and AS (*P* = 0.323 OR0.77; 95% CI = 0.47, 1.29) do not significantly share a genetic cause with AIT, according to the random-effects IVW study (**Figure 3**). Four supplementary analyses, as well as three validation approaches, consistently aligned with the outcomes of the IVW analysis (**Figures 4E, G** and **Supplementary Table 11**). The evaluation of genetic causality involving SS, or AS, in conjunction with AIT, exhibited uniformity in terms of the absence of heterogeneity (*P >*0.05), horizontal pleiotropy (*P >*0.05), and outliers (*P >*0.05) (**Supplementary Table 10**).

#### 3.2.6 Multiple sclerosis

The results of the IVW analysis with random effects showed no genetic causal connection between AIT and MS (*P* = 0.289, OR=0.94; 95% CI = 0.85,1.05) (**Figure 3**). However, it is noteworthy that Cochran’s Q statistic for the MR-IVW analysis indicated the presence of heterogeneity within the MS and AIT analysis (*P* < 0.05). Therefore, we used a random effects model to recalculate and obtained new results (*P* = 0.289, OR=0.94; 95% CI = 0.85,1.05). Four supplementary analyses consistently aligned with the outcomes of the random-effects IVW analysis (**Figure 4H**, **Supplementary Table 11**). There was no horizontal pleiotropy (*P >*0.05) or MR-PRESSO test outliers (*P >*0.05) in assessing the genetic causality of MS with AIT (**Supplementary Table 10**).

### 3.3 Results of the sensitivity analysis

**Figure 5:**
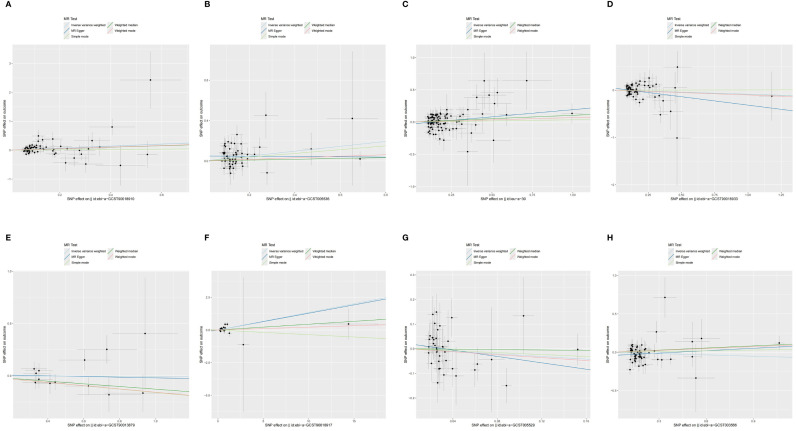
The funnel diagram of MR analysis between exposures (RA, T1D, CD, UC, SS, SLE, AS, and MS) and outcome (AIT). **(A)** RA and AIT; **(B)** T1D and AIT; **(C)** CD and AIT; **(D)** UC and AIT; **(E)** SS and AIT; **(F)** SLE and AIT; **(G)** AS and AIT; **(H)** MS and AIT.

**Figure 6:**
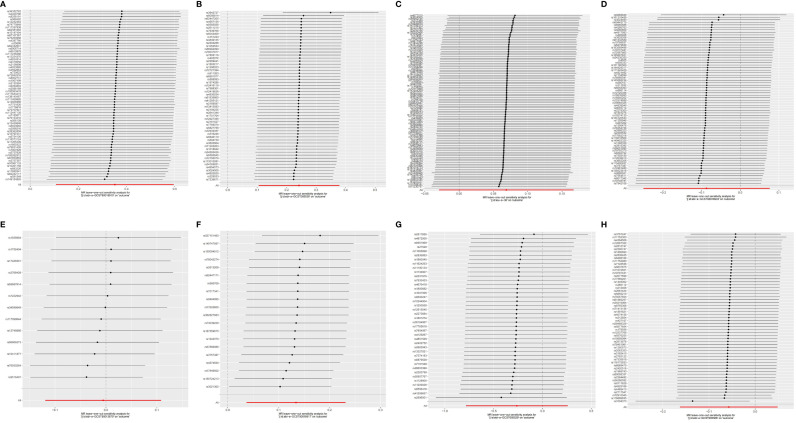
The leave-one-out analysis between exposures (RA, T1D, CD, UC, SS, SLE, AS, and MS) and outcome (AIT). **(A)** RA and AIT; **(B)** T1D and AIT; **(C)** CD and AIT; **(D)** UC and AIT; **(E)** SS and AIT; **(F)** SLE and AIT; **(G)** AS and AIT; **(H)** MS and AIT.

Pleiotropy in our study was assessed by examining the MR-Egger regression intercept and conducting the MR-PRESSO test. Heterogeneity within the dataset was evaluated using Cochran’s Q statistic. Detailed results from these tests are provided in **Supplementary Table 10**. Funnel plots showed that the distribution of IVs was symmetrical and did not indicate any significant abnormalities (**Figure 5**). The results of the leave-one-out analysis are shown in **Figure 6**, which suggests that the study had little potential bias because numerous SNPs did not cross the null line after being eliminated.

## 4 Discussion

This study aims to investigate the presence of a genetic causal interaction among common autoimmune diseases (AS, RA, SS, SLE, MS, T1D, UC, CD, and AIT). Using a two-sample MR analysis, compelling evidence supports a positive genetic causal relationship between RA, T1D, SLE, and AIT, suggesting these diseases may act as triggers for AIT. However, no significant positive or negative genetic causal relationship was found between AIT and other autoimmune diseases (AS, SS, MS, CD, and UC).

RA is characterized by polyarthritis, involving symmetrical joint inflammation in the small joints of the hands and feet, and often extends to affect organs and tissues outside the joints. Clinically, RA is identified by the presence of autoantibodies such as Rheumatoid Factor (RF) and Anti-citrullinated Protein Antibodies (ACPA) in the serum (23). A 2015 meta-analysis (24) of 1,021 RA patients and 1,500 healthy controls revealed a significantly higher prevalence of TG-Ab and TPO-Ab positivity in RA patients compared to healthy controls (25). This finding supports the higher prevalence of thyroid autoimmunity in RA patients relative to the general population. Additionally, TPO-Ab-positive RA patients are more likely to develop symptoms such as fibromyalgia or chronic generalized pain (26). Several studies have proposed an association between specific genetic loci and susceptibility to RA and AIT. HLA-DRB1 has been identified as the major susceptibility locus for RA (27). Arginine at position 74 of the HLA-DRB1 chain (DRB1 Arg74) is strongly associated with AIT susceptibility, which may be a key mechanism for the co-morbidity between RA and AIT (28). Furthermore, non-receptor-type protein tyrosine phosphatase 22 (PTPN22) is regulated by rs2476601 and plays an important role as a key regulator of the immune response (29). PTPN22 can inhibit citrullination, thereby enhancing peptidylarginine deaminase (PAD)-mediated hypercitrullination and hyperproliferation of T helper (Th) cells. This process can result in molecular features such as ACPA positivity in clinical RA patients (30). Previous studies have shown that normalized PTPN22 expression *in vitro* significantly correlates with the clinical outcomes of targeted therapies for RA, and circPTPN22 has potential as a diagnostic biomarker for RA (31, 32). Conversely, in AIT, the PTPN22 gene modulates the immune response by regulating the biological function of antigen-presenting cells, transcriptional repression of T lymphocytes, and differentiation and proliferation of B lymphocytes, thereby increasing the risk of developing AIT (33). Together, these findings suggest that PTPN22 may be a potential genetic link contributing to the co-morbidity between RA and AIT. Based on the results of this study, rs11889341 was identified as a significantly associated SNP for RA with AIT in the GWAS data of AIT. rs11889341 mediates the biological process of response to IL12 in lymphocytes and influences the differentiation of regulatory T cells (Tregs) by regulating the STAT4 gene (34). STAT4-mediated signal transduction is known to enhance the production of autoimmune-related components implicated in the pathogenesis of autoimmune diseases such as RA, SLE, and psoriasis (35). A study reported that the polymorphism of STAT4 (rs7574865/rs10181656) significantly increases the risk of AIT in the Chinese population (36). Additionally, these STAT4 alleles and the same haplotype may influence cytokine signaling and thereby contribute to the development of AITD (37). STAT4 is well-established as a risk factor for rheumatoid RA as well. Furthermore, we identified that the nuclear factor kappa B inhibitor epsilon (NFKBIE) rs28362859 is strongly correlated with RA susceptibility. NFKBIE, a core gene in the NF-κB pathway, has been associated significantly with RA, specifically cyclic citrullinated peptide (CCP)-positive RA and RF-positive RA (38, 39). These findings underscore the role of the NF-κB signaling pathway in the genetic pathogenesis of RA. Moreover, in various AITD, CD4 T cells interact with macrophages through the CXCR3-CXCL10/PKM-CD44/MHCII-NFKBIE axis. This interaction activates the T cell receptor signaling pathway, influences Th1 and Th2 cell differentiation, and modulates chemokine signaling pathways. Thus, NFKBIE plays a role in immune cell-mediated immune homeostasis, potentially exacerbating the immune response and directing immune cells to target thyroid tissues, thereby influencing disease progression in AITD (40). The study results establish a genetically driven causal relationship between RA and AIT. Therefore, patients diagnosed with RA should undergo close clinical monitoring for potential subsequent development of AIT.

T1D is a common form of AID in which the destruction of pancreatic β-cells mediated by the immune system leads to absolute insulin deficiency (41). There is growing evidence that patients with T1D have a very high prevalence of AIT, and T1D patients who are positive for multiple insulin autoantibodies are more likely to be positive for thyroid autoantibodies (42–44). Pathway analysis based on a genome-wide study of patients with both T1D and AIT implies that the signal transduction of cytotoxic T-lymphocyte antigen-4 (CTLA-4) and CD40 may be the principal pathway in their synergistic pathogenesis (45). Among these pathways, the CTLA-4 pathway is considered the main immunosuppressive axis in the body and serves as a crucial therapeutic target for enhancing anti-tumor immunity or inhibiting autoimmunity (46). In T1D, CTLA-4 can influence T cell-mediated immune regulation and play a pivotal role in the functional activity of Tregs, thereby inducing autoimmune responses in T1D (47). In AIT, CTLA-4, functioning as a regulatory factor mediating T cell tolerance and autoimmunity, exhibits dysfunction that indirectly results in an elevated secretion of thyroid autoantibodies. This dysfunction may be closely associated with the onset and progression of AIT (48). CD40 is known to play a crucial role in the T cell-mediated activation of B lymphocytes, participating in various immune and inflammatory responses (49). Previous studies have indicated that CD40-mediated signaling significantly enhances the production of pro-inflammatory cytokines, including Th1 and Th17, thereby influencing the development of T1D (50). Furthermore, CD40 signaling has been demonstrated to impact the production of pro-inflammatory cytokines and chemokines, contributing to thyroid gland tissue destruction and the infiltration of inflammatory cells, thereby increasing the susceptibility to AIT (51). The results of our study showed a significant correlation between rs12927355 in SNP and the development of T1D. Rs12927355 is involved in the regulation of the C-type lectin-like domain family 16A (CLEC16A), which has been identified as a disease susceptibility gene for T1D, MS, and adrenal dysfunction (52). Additionally, the CLEC16A locus rs3194051 has a significant impact on the prevalence of AITD in children (53). Therefore, we believe that CLEC16A rs12927355 may be significantly associated with genetic susceptibility to AIT and T1D. Furthermore, this study found that rs2304256 plays an important role in the pathogenesis of both conditions. For example, the A/A genotype of Tyrosine kinase 2 (TYK2) rs2304256 was associated with protection against T1D in a southern Brazilian population (54). TYK2 has also been identified as a pivotal gene in AITD, being greatly involved in genetic susceptibility pathways such as Th17 cell differentiation, and Th1 and Th2 cell differentiation. Thus, targeting TYK2 may be a potential avenue for future therapeutic improvement of AITD and its comorbidities (55). Combined with the results of this study, we believe that T1D is also a predisposing factor for AIT. Therefore, the expected susceptibility to AIT should be appropriately acknowledged in patients with T1D. T1D patients who are positive for multiple insulin autoantibodies should be routinely screened for thyroid antibodies to aid in the early diagnosis of AIT.

SLE is an autoimmune inflammatory connective tissue disease with a wide range of clinical manifestations (56). The results of several studies have shown that the risk associated with AIT is at least twice as high in patients with SLE compared to the healthy population. Additionally, the prevalence of thyroid cancer is higher in patients with SLE, particularly in the presence of autoimmune inflammation of the thyroid gland (57, 58). In this study, SNP rs960709 was found to be significantly associated with SLE in GWAS data related to AIT. SNP rs960709 regulates the expression of TNFAIP3 Interacting Protein 1 (TNIP1), which plays a crucial role in autoimmunity and tissue homeostasis by encoding an A20-binding protein. A20 is also a key regulator in inflammatory signaling pathways (59). It has been recognized that single nucleotide polymorphisms and haplotypes of TNIP1 are closely associated with the development of SLE in Chinese Han populations. Furthermore, it has been suggested that TNIP1 is a common SLE susceptibility gene shared by both Caucasian and Asian populations, and that its genetic contribution is greater in Japanese and Chinese populations (60, 61). In conjunction with the association of TNIP1 with TNFAIP3, numerous studies have highlighted their relevance to the NF-kb pathway, which is deeply involved in the onset and development of SLE (62, 63). Although studies related to the role of the TNIP1 gene in the pathogenesis of AIT are limited, it has been suggested that HT is one of the possible phenotypes of dysfunction in A20 haplogroups and that TNFAIP3 may be one of the genetic susceptibility factors for certain AITD (64, 65). Combined with the above studies, we believe that for SLE patients, testing of thyroid function and its antibodies, as well as relevant thyroid ultrasound, should be part of their routine clinical examination. SLE patients with high-risk factors, such as young women, combined with positive thyroid antibodies or concomitant hypoechoic thyroid nodules, should be followed up with thyroid function tests and provided with appropriate symptomatic supportive therapy.

In addition, some studies have identified potential correlations between AS, SS, MS, and the incidence of AIT. For instance, a cross-sectional study revealed a notable rise in the incidence of AITD among AS patients compared to the general population (66). This pathological characteristic is similarly observed in populations with SS (67), and MS (68). Therefore, many studies have delved into the potential pathophysiological mechanisms linking AIDs and AIT as mentioned above (69). Current research generally suggests that common genetic susceptibility and environmental factors play crucial roles in patients with AIDs and AIT. For instance, studies have validated that the functional single nucleotide polymorphism (rs2476601, encoding R620W) of PTPN22 can indicate susceptibility to four autoimmune phenotypes: T1D, RA, SLE, and AIT (70). Additionally, a prevalent subtype of Th1 has been identified in AIT, and its significance in the onset and progression of AIT has garnered recognition from numerous researchers (71). Th1 and its chemokines also exhibit a robust presence in AIDs such as AS (72), SS (73), and MS (74). However, research regarding the correlation between UC, CD, and AIT is relatively limited, with only a few case reports suggesting a potential association between UC, CD, and AIT. Nonetheless, some studies have proposed that the host’s immune system reacts to microbial abnormalities at the intestinal mucosal level, which could be a significant factor contributing to inflammatory bowel diseases such as UC and CD (75, 76). These inflammatory immune responses may subsequently extend to other organs and systems beyond the gastrointestinal tract, including the thyroid, ultimately resulting in the onset of AIT (77). In summary, while this study did not identify a causal relationship between AS, SS, MS, UC, CD, and AIT at the genetic level, the exclusion of genetic influence does not rule out the possibility of a potential association between these AIDs and AIT. Moreover, given the significant clinical ramifications of thyroid dysfunction, it remains imperative to recommend regular screening of thyroid function and antibodies for patients with either single or multiple AIDs in clinical practice.

However, the MR analysis in this study has several limitations. Firstly, the inclusion of genomic data from only European cohorts limits the ethnic diversity of the study. Future investigations should broaden the scope to include other ethnic groups with greater genetic diversity for further exploration and validation. Secondly, the scope of this study is limited to investigating the causal relationships between the eight common AIDs and AIT, without encompassing other potential associations between additional AIDs and AIT. In summary, our research findings provide genetic evidence for a potential causal link between AIT and RA, T1D, and SLE in the European population. These findings guide us to be vigilant about thyroid function and antibody levels in clinical practice for RA, T1D, and SLE patients.

## Data availability statement

The original contributions presented in the study are included in the article/**Supplementary Material**. Further inquiries can be directed to the corresponding author.

## Author contributions

KZ: Writing – review & editing, Writing – original draft, Data curation, Conceptualization. ZL: Writing – review & editing, Writing – original draft, Conceptualization. XW: Writing – review & editing, Writing – original draft, Supervision, Software, Methodology, Conceptualization.
