# Supplementary material for: The association of common autoimmune diseases with autoimmune thyroiditis: a two-sample Mendelian randomization study
Source: Front Endocrinol (Lausanne). 2024 Sep 9;15:1383221. doi: 10.3389/fendo.2024.1383221 (PMC11416997; doi:10.3389/fendo.2024.1383221)
Supplement: Supplementary file 1 [file Table_1.doc]

**Supplementary Table 1:** The data used in this study.

| **GWAS ID** | **Trait** | **Cases** | **Controls** | **Population** |
| --- | --- | --- | --- | --- |
| finn-b-E4_THYROIDITAUTOIM | Autoimmune thyroiditis | 489 | 320703 | European |
| ebi-a-GCST005529 | Ankylosing spondylitis | 9069 | 22647 | European |
| ebi-a-GCST90018910 | Rheumatoid arthritis | 8255 | 409,001 | European |
| ebi-a-GCST90013879 | Sjogren's syndrome | NA | 407,746 | European |
| ebi-a-GCST90018917 | Systemic lupus erythematosus | 647 | 482,264 | European |
| ebi-a-GCST003566 | multiple sclerosis | 4888 | 10395 | European |
| ebi-a-GCST005536 | Type 1 diabetes | 6683 | 12173 | European |
| ebi-a-GCST90018933 | Ulcerative colitis | 5371 | 432561 | European |
| ieu-a-30 | Crohn's disease | 5956 | 14927 | European |

**Supplementary Table 2:** The instrumental variables used in MR analysis between exposure (Rheumatoid arthritis) and outcome (Autoimmune thyroiditis).

|  | **SNP** | **beta.exposure** | **se.exposure** | **pval.exposure** | **pval.outcome** | **palindromic** | **F** |
| --- | --- | --- | --- | --- | --- | --- | --- |
| 1 | rs10147494 | 0.0811 | 0.0161 | 8.07E-07 | 0.436509 | FALSE | 25.37405964 |
| 2 | rs10261758 | -0.081 | 0.0143 | 5.48E-06 | 0.0790387 | FALSE | 32.08469852 |
| 3 | rs1067499 | -0.1365 | 0.0299 | 1.34E-08 | 0.803198 | FALSE | 20.84120983 |
| 4 | rs10797431 | -0.074 | 0.0129 | 7.33E-06 | 0.375994 | FALSE | 32.90667628 |
| 5 | rs111236286 | 0.2771 | 0.0619 | 6.21E-07 | 0.970617 | FALSE | 20.03972482 |
| 6 | rs111469885 | -0.1235 | 0.0222 | 5.61E-06 | 0.735594 | FALSE | 30.94767064 |
| 7 | rs111844273 | 0.3124 | 0.0706 | 8.96E-06 | 0.925631 | FALSE | 19.57999823 |
| 8 | rs112447135 | -0.2036 | 0.0431 | 8.35E-06 | 0.531145 | FALSE | 22.31521148 |
| 9 | rs11265409 | 0.1723 | 0.0374 | 4.36E-13 | 0.128049 | FALSE | 21.22400555 |
| 10 | rs116073124 | 0.3267 | 0.0716 | 7.69E-06 | 0.249979 | FALSE | 20.81959248 |
| 11 | rs117337938 | 0.2845 | 0.0564 | 6.19E-06 | 0.514099 | FALSE | 25.44522723 |
| 12 | rs11889341 | 0.1232 | 0.0146 | 3.34E-06 | 0.065274 | FALSE | 71.20585476 |
| 13 | rs12232384 | 0.0775 | 0.0143 | 5.87E-138 | 0.150454 | FALSE | 29.37185192 |
| 14 | rs12459648 | 0.0906 | 0.02 | 1.73E-06 | 0.427196 | FALSE | 20.5209 |
| 15 | rs12606866 | 0.0657 | 0.0147 | 4.68E-06 | 0.647878 | FALSE | 19.97542691 |
| 16 | rs13254370 | 0.0588 | 0.0133 | 3.06E-06 | 0.598237 | FALSE | 19.54570637 |
| 17 | rs138140487 | 0.3569 | 0.0804 | 7.83E-08 | 0.718686 | FALSE | 19.70520686 |
| 18 | rs149184893 | 0.4053 | 0.0896 | 1.88E-07 | 0.00463639 | FALSE | 20.46148682 |
| 19 | rs1660322 | 0.0715 | 0.0137 | 1.63E-06 | 0.156439 | FALSE | 27.23773243 |
| 20 | rs1776616 | 0.0832 | 0.0139 | 2.41E-06 | 0.611589 | FALSE | 35.82754516 |
| 21 | rs182262512 | 0.5558 | 0.1201 | 3.60E-07 | 0.0142837 | FALSE | 21.41662686 |
| 22 | rs192993419 | -0.0799 | 0.0176 | 1.41E-06 | 0.979974 | FALSE | 20.60953642 |
| 23 | rs2088189 | 0.1763 | 0.0372 | 8.05E-07 | 0.778314 | FALSE | 22.46046508 |
| 24 | rs2098216 | 0.106 | 0.0228 | 5.54E-06 | 0.441299 | FALSE | 21.61434287 |
| 25 | rs2156698 | -0.0745 | 0.0131 | 3.07E-06 | 0.872144 | FALSE | 32.34222947 |
| 26 | rs231806 | 0.0716 | 0.0135 | 9.36E-07 | 0.0101172 | TRUE | 28.12927298 |
| 27 | rs2409780 | 0.101 | 0.0147 | 2.97E-06 | 0.24608 | FALSE | 47.20718219 |
| 28 | rs2476601 | -0.3076 | 0.0253 | 2.66E-06 | 9.17E-13 | FALSE | 147.8194629 |
| 29 | rs2647166 | 0.0681 | 0.0137 | 1.31E-06 | 0.726439 | TRUE | 24.70888167 |
| 30 | rs28362859 | 0.1465 | 0.0205 | 8.76E-06 | 0.428909 | FALSE | 51.07019631 |
| 31 | rs3093017 | -0.1179 | 0.0128 | 8.96E-06 | 0.266357 | TRUE | 84.84136963 |
| 32 | rs3130384 | 0.0622 | 0.0135 | 2.09E-11 | 0.997713 | FALSE | 21.22820302 |
| 33 | rs34352762 | -0.0678 | 0.0147 | 9.36E-06 | 0.0422338 | FALSE | 21.272803 |
| 34 | rs34536443 | -0.2367 | 0.0427 | 9.70E-06 | 0.882249 | TRUE | 30.7284579 |
| 35 | rs35139284 | 0.5387 | 0.0149 | 5.71E-07 | 0.866354 | FALSE | 1307.137922 |
| 36 | rs35666 | -0.0797 | 0.0179 | 7.60E-06 | 0.336643 | FALSE | 19.82488062 |
| 37 | rs3757387 | 0.1086 | 0.0147 | 4.00E-06 | 0.163786 | FALSE | 54.57892545 |
| 38 | rs4287780 | -0.072 | 0.0156 | 1.53E-09 | 0.47639 | FALSE | 21.30177515 |
| 39 | rs4410848 | 0.0736 | 0.0154 | 4.81E-06 | 0.0232477 | TRUE | 22.84095126 |
| 40 | rs4592664 | -0.0758 | 0.0162 | 5.72E-06 | 0.787586 | FALSE | 21.89315653 |
| 41 | rs4924514 | 0.0725 | 0.0142 | 4.50E-06 | 0.741076 | FALSE | 26.06749653 |
| 42 | rs4933865 | 0.4379 | 0.0974 | 1.80E-06 | 0.448594 | FALSE | 20.21305588 |
| 43 | rs56074741 | -0.0722 | 0.0157 | 4.00E-06 | 0.100227 | TRUE | 21.14828188 |
| 44 | rs566128 | -0.0632 | 0.0137 | 1.00E-200 | 0.455271 | FALSE | 21.28104854 |
| 45 | rs5754100 | 0.086 | 0.015 | 2.44E-06 | 0.309115 | FALSE | 32.87111111 |
| 46 | rs5757628 | 0.0966 | 0.0157 | 6.82E-07 | 0.182431 | FALSE | 37.85776299 |
| 47 | rs57967133 | -0.1085 | 0.0231 | 2.34E-10 | 0.0769308 | FALSE | 22.06152433 |
| 48 | rs58107865 | -0.1156 | 0.0233 | 7.01E-14 | 0.680041 | TRUE | 24.61522592 |
| 49 | rs591549 | -0.0915 | 0.018 | 1.34E-07 | 0.0125473 | FALSE | 25.84027778 |
| 50 | rs61850863 | 0.1011 | 0.0227 | 2.69E-06 | 0.130104 | FALSE | 19.83584001 |
| 51 | rs62242117 | 0.1322 | 0.0285 | 1.93E-10 | 0.00725922 | FALSE | 21.51657741 |
| 52 | rs62342601 | 0.0925 | 0.0195 | 4.56E-06 | 0.707879 | FALSE | 22.50164366 |
| 53 | rs62381662 | 0.2227 | 0.0501 | 3.53E-13 | 0.128518 | FALSE | 19.75900096 |
| 54 | rs67167367 | -0.1133 | 0.0211 | 4.85E-27 | 0.582648 | FALSE | 28.83333708 |
| 55 | rs6906714 | 0.2289 | 0.0182 | 3.85E-06 | 0.535502 | FALSE | 158.1789941 |
| 56 | rs6910879 | 0.5436 | 0.0401 | 9.03E-07 | 0.747443 | FALSE | 183.7681109 |
| 57 | rs6924682 | 0.0879 | 0.0176 | 1.99E-23 | 0.415885 | FALSE | 24.9432141 |
| 58 | rs6930052 | -0.2124 | 0.0352 | 6.16E-06 | 0.662354 | TRUE | 36.4102531 |
| 59 | rs7089017 | -0.0702 | 0.0154 | 6.23E-12 | 0.381427 | TRUE | 20.77938944 |
| 60 | rs71508903 | 0.0872 | 0.0157 | 7.10E-07 | 0.70732 | FALSE | 30.84847255 |
| 61 | rs7180071 | -0.0656 | 0.014 | 1.75E-06 | 0.508761 | FALSE | 21.95591837 |
| 62 | rs720622 | -0.0633 | 0.0141 | 4.43E-06 | 0.735005 | FALSE | 20.15436849 |
| 63 | rs72707941 | 0.0761 | 0.0165 | 4.84E-13 | 0.595976 | FALSE | 21.27166208 |
| 64 | rs72842516 | -0.0765 | 0.0173 | 2.00E-06 | 0.929287 | FALSE | 19.55377727 |
| 65 | rs74625075 | -0.1291 | 0.0282 | 1.24E-06 | 0.124624 | TRUE | 20.95821387 |
| 66 | rs74770848 | -0.1843 | 0.041 | 9.96E-06 | 0.4572 | FALSE | 20.20612136 |
| 67 | rs76153210 | 0.1151 | 0.0232 | 1.46E-08 | 0.318297 | FALSE | 24.61357387 |
| 68 | rs76260988 | 0.2563 | 0.057 | 9.12E-06 | 0.346524 | FALSE | 20.21843336 |
| 69 | rs76631426 | -0.1159 | 0.0253 | 5.61E-06 | 0.0199127 | FALSE | 20.98581449 |
| 70 | rs7731626 | -0.1343 | 0.0157 | 3.69E-06 | 0.465088 | FALSE | 73.17331332 |
| 71 | rs78019882 | 0.1126 | 0.0215 | 1.56E-08 | 0.186399 | TRUE | 27.42836128 |
| 72 | rs7907829 | 0.0672 | 0.0133 | 7.39E-08 | 0.133018 | FALSE | 25.52908587 |
| 73 | rs8002731 | -0.0717 | 0.0137 | 1.51E-06 | 0.973916 | FALSE | 27.39032447 |
| 74 | rs883220 | -0.0822 | 0.0184 | 3.44E-06 | 0.0282514 | FALSE | 19.95758507 |
| 75 | rs9258191 | -0.1787 | 0.0214 | 1.13E-87 | 0.272254 | FALSE | 69.73030396 |
| 76 | rs9277398 | -0.121 | 0.0138 | 2.34E-09 | 0.000416946 | FALSE | 76.87985717 |
| 77 | rs9318056 | -0.071 | 0.0152 | 4.49E-07 | 0.765843 | FALSE | 21.81873269 |
| 78 | rs9494894 | 0.1726 | 0.031 | 2.31E-90 | 0.92213 | FALSE | 30.99975026 |
| 79 | rs988482 | 0.302 | 0.0679 | 2.61E-06 | 0.109572 | FALSE | 19.78218857 |
| 80 | rs10147494 | 0.0811 | 0.0161 | 3.47E-07 | 0.436509 | FALSE | 25.37405964 |
| Confounding | | | | | | | |
| 1 | rs71508903 | Hypothyroidism | | | | | |
| 2 | rs2476601 | Autoimmune thyroid diseases (Graves disease or Hashimoto's thyroiditis) | | | | | |

**Supplementary Table 3:** The instrumental variables used in MR analysis between exposure (Ankylosing spondylitis) and outcome (Autoimmune thyroiditis).

|  | **SNP** | beta.exposure | se.exposure | pval.exposure | pval.outcome | palindromic | F |
| --- | --- | --- | --- | --- | --- | --- | --- |
| 1 | rs10244904 | 0.0254728 | 0.00547054 | 3.22E-06 | 0.873288 | FALSE | 21.68168226 |
| 2 | rs1041926 | -0.0748291 | 0.0116918 | 1.55E-10 | 0.835952 | FALSE | 40.96172978 |
| 3 | rs11065898 | 0.0262524 | 0.00480641 | 4.71E-08 | 0.0862542 | FALSE | 29.83296349 |
| 4 | rs11190133 | -0.0338671 | 0.00449401 | 4.84E-14 | 0.464906 | FALSE | 56.79210306 |
| 5 | rs11209026 | -0.103584 | 0.00954481 | 1.94E-27 | 0.390528 | FALSE | 117.7743674 |
| 6 | rs1128905 | -0.0237165 | 0.00409459 | 6.95E-09 | 0.10806 | FALSE | 33.54905971 |
| 7 | rs11624293 | 0.0428679 | 0.00669161 | 1.49E-10 | 0.345313 | FALSE | 41.03960526 |
| 8 | rs1250550 | -0.026036 | 0.00430465 | 1.46E-09 | 0.910363 | FALSE | 36.5824539 |
| 9 | rs12615545 | 0.0254728 | 0.00417273 | 1.03E-09 | 0.980549 | FALSE | 37.26600201 |
| 10 | rs13327021 | 0.0218593 | 0.00423167 | 2.40E-07 | 0.506041 | FALSE | 26.68387611 |
| 11 | rs1734907 | -0.0282959 | 0.00584865 | 1.31E-06 | 0.327251 | FALSE | 23.40646122 |
| 12 | rs17765610 | 0.0356567 | 0.00655388 | 5.31E-08 | 0.808028 | FALSE | 29.59956466 |
| 13 | rs1800682 | -0.018164 | 0.00410687 | 9.74E-06 | 0.795794 | FALSE | 19.5614514 |
| 14 | rs1801274 | 0.0253178 | 0.00417687 | 1.35E-09 | 0.954091 | FALSE | 36.74091877 |
| 15 | rs1860545 | -0.027474 | 0.00435378 | 2.78E-10 | 0.207949 | FALSE | 39.82087767 |
| 16 | rs2273684 | 0.018331 | 0.00414588 | 9.80E-06 | 0.901298 | FALSE | 19.54964429 |
| 17 | rs2283790 | 0.0263498 | 0.00490734 | 7.90E-08 | 0.267662 | FALSE | 28.83117545 |
| 18 | rs2402752 | -0.022757 | 0.00451912 | 4.76E-07 | 0.632284 | FALSE | 25.35842473 |
| 19 | rs2517655 | 0.088194 | 0.00465884 | 6.40E-80 | 0.0349374 | FALSE | 358.3624437 |
| 20 | rs2531875 | -0.0273233 | 0.00424463 | 1.22E-10 | 0.442933 | FALSE | 41.43683945 |
| 21 | rs2596501 | -0.152336 | 0.00419401 | 1.00E-200 | 0.965007 | FALSE | 1319.307928 |
| 22 | rs2663043 | -0.0188218 | 0.00411076 | 4.68E-06 | 0.555564 | FALSE | 20.96418892 |
| 23 | rs27529 | -0.0620354 | 0.00429859 | 3.28E-47 | 0.354123 | FALSE | 208.270196 |
| 24 | rs2836883 | -0.0396768 | 0.00474798 | 6.46E-17 | 0.275439 | FALSE | 69.83216632 |
| 25 | rs28653390 | 0.0282959 | 0.00572812 | 7.82E-07 | 0.317223 | FALSE | 24.40185313 |
| 26 | rs35164067 | -0.031078 | 0.00495021 | 3.43E-10 | 0.930111 | FALSE | 39.41475927 |
| 27 | rs4129267 | -0.0307685 | 0.00422605 | 3.32E-13 | 0.90122 | FALSE | 53.00824023 |
| 28 | rs41299637 | -0.0390527 | 0.00491011 | 1.81E-15 | 0.106367 | FALSE | 63.25861617 |
| 29 | rs4672505 | -0.0597772 | 0.00415105 | 5.14E-47 | 0.205618 | FALSE | 207.3744313 |
| 30 | rs4676410 | 0.0281014 | 0.00490215 | 9.90E-09 | 0.690114 | FALSE | 32.86114726 |
| 31 | rs4851529 | -0.0225524 | 0.00423971 | 1.04E-07 | 0.747555 | FALSE | 28.29523001 |
| 32 | rs55957767 | -0.0219389 | 0.00455968 | 1.50E-06 | 0.0595676 | FALSE | 23.15053157 |
| 33 | rs6556416 | 0.0252152 | 0.0046002 | 4.22E-08 | 0.0420552 | FALSE | 30.04494457 |
| 34 | rs6600247 | 0.0328332 | 0.00415088 | 2.58E-15 | 0.839927 | FALSE | 62.56710603 |
| 35 | rs6879529 | -0.0216323 | 0.00421448 | 2.85E-07 | 0.485591 | FALSE | 26.34616455 |
| 36 | rs7191548 | 0.0249853 | 0.00428493 | 5.51E-09 | 0.516854 | FALSE | 34.00021582 |
| 37 | rs7374183 | 0.0194887 | 0.00417674 | 3.07E-06 | 0.430732 | FALSE | 21.77162562 |
| 38 | rs7933433 | 0.0206846 | 0.00426839 | 1.26E-06 | 0.416878 | FALSE | 23.48367526 |
| 39 | rs7954567 | 0.0239118 | 0.00452637 | 1.27E-07 | 0.84277 | FALSE | 27.90772506 |
| 40 | rs9901869 | 0.0319036 | 0.00408853 | 6.04E-15 | 0.0977485 | FALSE | 60.88986422 |

**Supplementary Table 4:** The instrumental variables used in MR analysis between exposure (Sjogren's syndrome) and outcome (Autoimmune thyroiditis).

|  | SNP | beta.exposure | se.exposure | pval.exposure | pval.outcome | palindromic | F |
| --- | --- | --- | --- | --- | --- | --- | --- |
| 1 | rs10411877 | 0.323226 | 0.0720603 | 9.09E-06 | 0.298555 | TRUE | 20.11965103 |
| 2 | rs117099644 | 0.942071 | 0.180972 | 6.54E-06 | 0.450692 | TRUE | 27.09845297 |
| 3 | rs12748886 | 0.334203 | 0.0713748 | 3.77E-06 | 0.711313 | TRUE | 21.92455965 |
| 4 | rs149449770 | 0.889814 | 0.15574 | 5.41E-07 | 0.121092 | TRUE | 32.64360607 |
| 5 | rs1556984 | -0.626082 | 0.133857 | 8.55E-07 | 0.23723 | TRUE | 21.87662191 |
| 6 | rs17426591 | -0.740768 | 0.178597 | 6.85E-06 | 0.263523 | TRUE | 17.2034721 |
| 7 | rs1752404 | -0.329943 | 0.0702081 | 4.31E-06 | 0.315582 | TRUE | 22.08530481 |
| 8 | rs1811197 | 0.604747 | 0.072278 | 1.20E-14 | 1.09E-05 | TRUE | 70.00598841 |
| 9 | rs2768428 | 0.440695 | 0.101855 | 8.70E-06 | 0.417564 | TRUE | 18.72024551 |
| 10 | rs34586649 | 0.349876 | 0.0758386 | 9.40E-06 | 0.708399 | TRUE | 21.28372756 |
| 11 | rs55687614 | -0.930092 | 0.212797 | 1.28E-06 | 0.355308 | TRUE | 19.10384632 |
| 12 | rs56666273 | -0.350435 | 0.0770479 | 5.38E-06 | 0.485671 | TRUE | 20.68680106 |
| 13 | rs7222942 | 0.411875 | 0.0846509 | 4.15E-06 | 0.471158 | TRUE | 23.67378528 |
| 14 | rs76550264 | -0.605001 | 0.143806 | 7.69E-06 | 0.12902 | TRUE | 17.69938483 |
| 15 | rs9516497 | -0.728772 | 0.167533 | 2.41E-06 | 0.0604686 | TRUE | 18.92267916 |

**Supplementary Table 5:** The instrumental variables used in MR analysis between exposure (Systemic lupus erythematosus) and outcome (Autoimmune thyroiditis).

|  | SNP | beta.exposure | se.exposure | pval.exposure | pval.outcome | palindromic | F |
| --- | --- | --- | --- | --- | --- | --- | --- |
| 1 | rs115526225 | 0.8337 | 0.1861 | 7.48E-06 | 0.609434 | TRUE | 20.06905193 |
| 2 | rs1243370 | -0.4547 | 0.1019 | 8.07E-06 | 0.397406 | FALSE | 19.91138824 |
| 3 | rs149747957 | 1.2567 | 0.281 | 7.75E-06 | 0.344505 | FALSE | 20.00094844 |
| 4 | rs150094912 | 0.2897 | 0.0612 | 2.21E-06 | 0.205257 | FALSE | 22.40753823 |
| 5 | rs150724213 | 1.0244 | 0.2228 | 4.27E-06 | 0.00788061 | FALSE | 21.14018417 |
| 6 | rs17625605 | 0.7312 | 0.1609 | 5.51E-06 | 0.944505 | FALSE | 20.65191254 |
| 7 | rs17849502 | 0.7862 | 0.14 | 1.95E-08 | 0.010335 | FALSE | 31.53624694 |
| 8 | rs187559875 | 0.7877 | 0.1725 | 4.99E-06 | 0.482485 | FALSE | 20.85179719 |
| 9 | rs2613208 | -0.2266 | 0.0473 | 1.63E-06 | 0.638463 | FALSE | 22.95078421 |
| 10 | rs3021302 | 0.6262 | 0.0724 | 5.25E-18 | 0.00721257 | FALSE | 74.80816367 |
| 11 | rs371642845 | 1.7183 | 0.3427 | 5.32E-07 | 0.598159 | TRUE | 25.14026038 |
| 12 | rs3757387 | 0.2685 | 0.0519 | 2.30E-07 | 0.163786 | FALSE | 26.76417521 |
| 13 | rs4274624 | -0.3778 | 0.0529 | 9.37E-13 | 0.0630406 | FALSE | 51.00497783 |
| 14 | rs4642071 | 0.3376 | 0.072 | 2.74E-06 | 0.862219 | TRUE | 21.98567901 |
| 15 | rs537151493 | 14.3938 | 3.2386 | 8.81E-06 | 0.690613 | FALSE | 19.75314741 |
| 16 | rs539937225 | 1.5985 | 0.3581 | 8.07E-06 | 0.654012 | TRUE | 19.92584002 |
| 17 | rs562827883 | 0.5064 | 0.1136 | 8.34E-06 | 0.830258 | FALSE | 19.87150367 |
| 18 | rs57880986 | 0.3452 | 0.0774 | 8.12E-06 | 0.389285 | FALSE | 19.89113902 |
| 19 | rs62447171 | -0.2264 | 0.0512 | 9.71E-06 | 0.76055 | FALSE | 19.55297852 |
| 20 | rs7017341 | -0.2931 | 0.0629 | 3.11E-06 | 0.999552 | FALSE | 21.71352565 |
| 21 | rs72712464 | 0.3258 | 0.0732 | 8.55E-06 | 0.327091 | TRUE | 19.80979575 |
| 22 | rs73039228 | 2.7976 | 0.6319 | 9.53E-06 | 0.827824 | FALSE | 19.60083297 |
| 23 | rs77063044 | 0.5375 | 0.1069 | 4.92E-07 | 0.969206 | TRUE | 25.28142367 |
| 24 | rs78042274 | 0.6971 | 0.153 | 5.25E-06 | 0.769309 | FALSE | 20.75904182 |
| 25 | rs9494895 | 0.6663 | 0.1193 | 2.31E-08 | 0.849215 | FALSE | 31.19311449 |
| 26 | rs960709 | 0.2422 | 0.0521 | 3.35E-06 | 0.952468 | FALSE | 21.61089887 |

**Supplementary Table 6:** The instrumental variables used in MR analysis between exposure (Multiple Sclerosis) and outcome (Autoimmune thyroiditis).

|  | **SNP** | **beta.exposure** | **se.exposure** | **pval.exposure** | **pval.outcome** | **palindromic** | **F** |
| --- | --- | --- | --- | --- | --- | --- | --- |
| 1 | rs10797431 | beta.exposure | 0.0269989 | 1.81E-10 | 0.375994 | FALSE | 40.66093982 |
| 2 | rs10896028 | 0.172161 | 0.0257575 | 2.25E-07 | 0.973243 | TRUE | 26.80391078 |
| 3 | rs10999984 | -0.133353 | 0.0456066 | 5.04E-06 | 0.779403 | FALSE | 20.82138606 |
| 4 | rs11060994 | 0.208105 | 0.046072 | 9.17E-06 | 0.737498 | TRUE | 19.67628744 |
| 5 | rs116175833 | -0.204366 | 0.109251 | 2.81E-06 | 0.592178 | FALSE | 21.94519977 |
| 6 | rs11672651 | -0.511794 | 0.0476799 | 8.16E-06 | 0.184691 | FALSE | 19.89920482 |
| 7 | rs116899835 | 0.212693 | 0.0617958 | 4.50E-06 | 0.0593759 | FALSE | 21.03918324 |
| 8 | rs11752303 | 0.283448 | 0.029883 | 4.50E-36 | 0.175397 | FALSE | 157.2567868 |
| 9 | rs11752980 | 0.374739 | 0.0313184 | 3.33E-06 | 0.152922 | FALSE | 21.61342616 |
| 10 | rs117861023 | 0.1456 | 0.0938359 | 2.87E-07 | 0.823811 | TRUE | 26.33350401 |
| 11 | rs1250573 | -0.48153 | 0.0272206 | 1.64E-06 | 0.714404 | FALSE | 22.98012668 |
| 12 | rs12537766 | -0.130489 | 0.0279089 | 8.48E-06 | 0.889012 | FALSE | 19.82750764 |
| 13 | rs12697352 | 0.124273 | 0.0271219 | 1.37E-06 | 0.0391201 | FALSE | 23.32221268 |
| 14 | rs1445002 | 0.13098 | 0.0351095 | 1.14E-07 | 0.433676 | FALSE | 28.12825024 |
| 15 | rs17569201 | -0.186207 | 0.0301992 | 6.58E-07 | 0.4041 | FALSE | 24.73513533 |
| 16 | rs179939 | 0.150194 | 0.0366337 | 7.98E-06 | 0.991071 | FALSE | 19.94318009 |
| 17 | rs1800693 | -0.163598 | 0.0252103 | 1.06E-09 | 0.199615 | FALSE | 37.21540569 |
| 18 | rs1891621 | -0.153794 | 0.0255751 | 2.94E-08 | 0.779119 | FALSE | 30.74662195 |
| 19 | rs1969743 | 0.141813 | 0.0250566 | 1.84E-06 | 0.452328 | FALSE | 22.75671439 |
| 20 | rs2063353 | -0.11953 | 0.0250836 | 1.16E-06 | 0.675464 | FALSE | 23.64855698 |
| 21 | rs2077599 | 0.121981 | 0.0309763 | 2.02E-06 | 0.298612 | FALSE | 22.57371844 |
| 22 | rs212408 | 0.147174 | 0.0275047 | 9.39E-08 | 0.447443 | FALSE | 28.49576224 |
| 23 | rs2182410 | -0.146824 | 0.0262643 | 1.15E-11 | 0.806717 | FALSE | 46.05830215 |
| 24 | rs2300747 | -0.178246 | 0.0414406 | 1.74E-12 | 0.25591 | FALSE | 49.76232304 |
| 25 | rs2364482 | 0.292332 | 0.0297593 | 5.86E-08 | 0.337347 | FALSE | 29.41050206 |
| 26 | rs2420518 | -0.161389 | 0.0253684 | 6.50E-07 | 0.520994 | FALSE | 24.7569833 |
| 27 | rs2513676 | 0.126224 | 0.0271314 | 9.56E-06 | 0.726653 | FALSE | 19.59777168 |
| 28 | rs2681424 | 0.120109 | 0.0252049 | 9.51E-10 | 0.56124 | FALSE | 37.42284718 |
| 29 | rs2717547 | 0.154189 | 0.0281613 | 2.92E-06 | 0.179747 | FALSE | 21.86888192 |
| 30 | rs2763122 | -0.131694 | 0.0313708 | 1.18E-06 | 0.647138 | FALSE | 23.60253449 |
| 31 | rs2812197 | -0.152407 | 0.0259613 | 9.95E-09 | 0.0738822 | FALSE | 32.85177413 |
| 32 | rs2836425 | 0.148801 | 0.0357948 | 2.84E-08 | 0.113007 | FALSE | 30.81207046 |
| 33 | rs28488329 | -0.198692 | 0.0260386 | 1.90E-06 | 0.97988 | FALSE | 22.69171118 |
| 34 | rs3104373 | -0.124037 | 0.0325572 | 1.00E-200 | 0.189617 | FALSE | 1069.590453 |
| 35 | rs34286592 | 1.06477 | 0.0348499 | 4.58E-08 | 0.329547 | FALSE | 29.88545256 |
| 36 | rs35219064 | -0.190516 | 0.0376961 | 2.24E-06 | 0.574101 | FALSE | 22.37752959 |
| 37 | rs360112 | 0.178321 | 0.0351229 | 6.34E-08 | 0.407515 | FALSE | 29.25581304 |
| 38 | rs3757247 | -0.189975 | 0.024837 | 3.29E-06 | 0.000398089 | FALSE | 21.64045819 |
| 39 | rs4074156 | -0.11554 | 0.0388364 | 6.38E-06 | 0.858526 | FALSE | 20.37189344 |
| 40 | rs411919 | -0.175289 | 0.0282663 | 6.26E-08 | 0.000364838 | FALSE | 29.27970613 |
| 41 | rs4364506 | -0.152951 | 0.0292415 | 4.06E-09 | 0.014288 | FALSE | 34.59530786 |
| 42 | rs437107 | 0.171992 | 0.0386501 | 1.70E-06 | 0.941939 | FALSE | 22.90842655 |
| 43 | rs4469413 | -0.18499 | 0.0279977 | 6.35E-06 | 0.20641 | FALSE | 20.3801889 |
| 44 | rs4525910 | 0.126394 | 0.0251906 | 9.74E-06 | 0.938174 | TRUE | 19.56186717 |
| 45 | rs4925166 | -0.111415 | 0.0267995 | 2.69E-09 | 0.377042 | FALSE | 35.39893668 |
| 46 | rs512854 | -0.159449 | 0.0327639 | 1.06E-07 | 0.871271 | FALSE | 28.26573228 |
| 47 | rs61885257 | 0.174191 | 0.118954 | 7.00E-06 | 0.44527 | FALSE | 20.19457956 |
| 48 | rs6498168 | -0.53456 | 0.0258145 | 1.98E-15 | 0.309652 | FALSE | 63.08351162 |
| 49 | rs6689470 | 0.205032 | 0.0343181 | 3.93E-10 | 0.62444 | FALSE | 39.14590974 |
| 50 | rs6755306 | -0.214717 | 0.0290923 | 2.69E-07 | 0.663593 | FALSE | 26.45939532 |
| 51 | rs6837875 | -0.149647 | 0.0617092 | 2.62E-07 | 0.363203 | FALSE | 26.51475919 |
| 52 | rs6859219 | -0.317756 | 0.0312555 | 8.06E-09 | 0.567673 | FALSE | 33.25996125 |
| 53 | rs71413156 | 0.180255 | 0.0382648 | 3.27E-06 | 0.729778 | FALSE | 21.65013798 |
| 54 | rs7145536 | -0.178045 | 0.0250521 | 2.53E-06 | 0.14153 | FALSE | 22.1467431 |
| 55 | rs72910045 | 0.117896 | 0.0730092 | 3.73E-06 | 0.00613437 | FALSE | 21.39955169 |
| 56 | rs73720868 | 0.337738 | 0.0519161 | 8.59E-06 | 0.440939 | TRUE | 19.80118944 |
| 57 | rs75057504 | -0.231019 | 0.0567847 | 2.03E-19 | 0.660011 | FALSE | 81.20575984 |
| 58 | rs7535818 | 0.511711 | 0.0339461 | 1.51E-15 | 0.833542 | FALSE | 63.62499088 |
| 59 | rs767626 | -0.270772 | 0.0368254 | 7.25E-06 | 0.845813 | TRUE | 20.12645106 |
| 60 | rs78451961 | -0.165208 | 0.0341194 | 2.84E-07 | 0.820278 | FALSE | 26.35253942 |
| 61 | rs79638626 | 0.175151 | 0.0525243 | 8.04E-06 | 0.372618 | TRUE | 19.92772516 |
| 62 | rs80008337 | -0.234471 | 0.118409 | 1.46E-06 | 0.479809 | FALSE | 23.2031511 |
| 63 | rs8111628 | -0.570372 | 0.0284534 | 3.89E-06 | 0.287998 | FALSE | 21.32014611 |
| 64 | rs9277654 | 0.13138 | 0.0339235 | 3.33E-12 | 0.923836 | FALSE | 48.48610695 |
| 65 | rs9376255 | 0.236216 | 0.0259245 | 1.01E-06 | 0.864973 | FALSE | 23.90010309 |

**Supplementary Table 7:** The instrumental variables used in MR analysis between exposure (Type 1 diabetes) and outcome (Autoimmune thyroiditis).

|  | SNP | beta.exposure | se.exposure | pval.exposure | pval.outcome | palindromic | F |
| --- | --- | --- | --- | --- | --- | --- | --- |
| 1 | rs1018942 | 0.272991 | 0.0529982 | 2.59E-07 | 0.0478311 | FALSE | 26.53227068 |
| 2 | rs1052553 | -0.117996 | 0.0219992 | 8.16E-08 | 0.288995 | FALSE | 28.76873689 |
| 3 | rs11203203 | 0.149971 | 0.0189963 | 2.91E-15 | 0.160308 | FALSE | 62.32704479 |
| 4 | rs113010081 | -0.16405 | 0.0300091 | 4.59E-08 | 0.0781466 | FALSE | 29.88453674 |
| 5 | rs12150079 | 0.114043 | 0.0200075 | 1.20E-08 | 0.0796581 | FALSE | 32.49014245 |
| 6 | rs12416116 | -0.164993 | 0.0209991 | 3.93E-15 | 0.645565 | FALSE | 61.73474733 |
| 7 | rs12418638 | 0.104 | 0.0219999 | 2.28E-06 | 0.735221 | FALSE | 22.34731059 |
| 8 | rs12927355 | -0.192008 | 0.0200009 | 7.99E-22 | 0.366863 | FALSE | 92.15938563 |
| 9 | rs12932357 | -0.140979 | 0.0289956 | 1.16E-06 | 0.352748 | FALSE | 23.63984646 |
| 10 | rs1296023 | -0.0939811 | 0.019996 | 2.60E-06 | 0.984044 | FALSE | 22.08995299 |
| 11 | rs13415583 | -0.101037 | 0.0190069 | 1.06E-07 | 0.507281 | FALSE | 28.25779731 |
| 12 | rs1456988 | -0.111005 | 0.0200008 | 2.86E-08 | 0.748809 | FALSE | 30.80281079 |
| 13 | rs151233 | 0.171008 | 0.0260012 | 4.80E-11 | 0.595088 | FALSE | 43.25597164 |
| 14 | rs1534422 | -0.0839734 | 0.018994 | 9.82E-06 | 0.030153 | FALSE | 19.54567207 |
| 15 | rs1574285 | -0.115024 | 0.0190039 | 1.42E-09 | 0.809936 | FALSE | 36.63460047 |
| 16 | rs1701704 | 0.222984 | 0.0189986 | 8.25E-32 | 0.339247 | FALSE | 137.7539958 |
| 17 | rs1893217 | 0.192024 | 0.024003 | 1.24E-15 | 0.821191 | FALSE | 64 |
| 18 | rs2111485 | 0.164993 | 0.0189991 | 3.81E-18 | 0.287599 | FALSE | 75.41625824 |
| 19 | rs2168587 | 0.167969 | 0.0339937 | 7.77E-07 | 0.535802 | FALSE | 24.41526253 |
| 20 | rs2181527 | 0.0879573 | 0.0189908 | 3.63E-06 | 0.350829 | FALSE | 21.4514799 |
| 21 | rs2194225 | 0.103008 | 0.0190014 | 5.92E-08 | 0.471863 | FALSE | 29.38804588 |
| 22 | rs2269241 | 0.115024 | 0.0230047 | 5.73E-07 | 0.99014 | FALSE | 25.00021735 |
| 23 | rs229533 | 0.106969 | 0.0189945 | 1.79E-08 | 0.00252773 | FALSE | 31.71466584 |
| 24 | rs2304256 | -0.139032 | 0.0210049 | 3.61E-11 | 0.737401 | FALSE | 43.81151828 |
| 25 | rs2611215 | -0.167969 | 0.0249954 | 1.82E-11 | 0.779636 | FALSE | 45.15835268 |
| 26 | rs2641348 | 0.144014 | 0.0290028 | 6.85E-07 | 0.46231 | FALSE | 24.65639454 |
| 27 | rs3024505 | -0.145951 | 0.0269909 | 6.39E-08 | 0.00448291 | FALSE | 29.24013797 |
| 28 | rs3087243 | -0.178051 | 0.0190054 | 7.36E-21 | 0.0109187 | FALSE | 87.76771775 |
| 29 | rs3184504 | -0.266051 | 0.0190036 | 1.56E-44 | 0.000972837 | FALSE | 196.000884 |
| 30 | rs34185821 | 0.0910283 | 0.0200062 | 5.36E-06 | 0.0300027 | FALSE | 20.70254094 |
| 31 | rs34593439 | -0.246028 | 0.0330038 | 9.02E-14 | 0.154192 | FALSE | 55.57010009 |
| 32 | rs3802604 | -0.0910283 | 0.0190059 | 1.67E-06 | 0.199582 | FALSE | 22.93907939 |
| 33 | rs3842727 | 0.686966 | 0.0229989 | 4.89E-196 | 0.792388 | FALSE | 892.1879561 |
| 34 | rs402072 | -0.142025 | 0.0260045 | 4.72E-08 | 0.932417 | FALSE | 29.82858008 |
| 35 | rs41295121 | -0.652965 | 0.111994 | 5.53E-09 | 0.526588 | FALSE | 33.99306255 |
| 36 | rs4820830 | -0.134968 | 0.0189955 | 1.20E-12 | 0.00899725 | FALSE | 50.48474486 |
| 37 | rs4849135 | 0.114962 | 0.0209931 | 4.35E-08 | 0.472769 | FALSE | 29.98855034 |
| 38 | rs4930034 | 0.0910194 | 0.0190041 | 1.67E-06 | 0.709151 | FALSE | 22.93893879 |
| 39 | rs4954573 | 0.0930349 | 0.0210079 | 9.49E-06 | 0.0138475 | FALSE | 19.61220876 |
| 40 | rs4957135 | -0.0890177 | 0.020004 | 8.59E-06 | 0.272223 | FALSE | 19.80245551 |
| 41 | rs516246 | 0.142947 | 0.018993 | 5.22E-14 | 0.344561 | FALSE | 56.64517909 |
| 42 | rs56994090 | -0.12897 | 0.0189956 | 1.13E-11 | 0.899926 | FALSE | 46.09686261 |
| 43 | rs6043409 | 0.126017 | 0.0200027 | 2.98E-10 | 0.623733 | FALSE | 39.6899937 |
| 44 | rs61839660 | -0.471925 | 0.0359943 | 2.84E-39 | 0.462121 | FALSE | 171.9010403 |
| 45 | rs62447205 | -0.116983 | 0.020997 | 2.53E-08 | 0.429885 | FALSE | 31.0406647 |
| 46 | rs6592645 | 0.0950374 | 0.0190075 | 5.73E-07 | 0.0468781 | FALSE | 24.99994739 |
| 47 | rs6679677 | 0.635995 | 0.0269998 | 1.10E-122 | 5.44E-13 | FALSE | 554.8636935 |
| 48 | rs6691977 | 0.126016 | 0.0230029 | 4.30E-08 | 0.804814 | FALSE | 30.011396 |
| 49 | rs6827756 | -0.131028 | 0.0190041 | 5.40E-12 | 0.361888 | FALSE | 47.53720135 |
| 50 | rs6840119 | -0.0920321 | 0.020007 | 4.22E-06 | 0.232843 | FALSE | 21.15995402 |
| 51 | rs694739 | -0.0850134 | 0.019003 | 7.69E-06 | 0.210354 | FALSE | 20.0138405 |
| 52 | rs7239671 | 0.120978 | 0.0179967 | 1.79E-11 | 0.00467391 | FALSE | 45.18840856 |
| 53 | rs72727394 | 0.138021 | 0.0220034 | 3.55E-10 | 0.83511 | FALSE | 39.34692084 |
| 54 | rs72928038 | 0.179985 | 0.0239981 | 6.38E-14 | 0.13397 | FALSE | 56.24953121 |
| 55 | rs7795074 | 0.0900341 | 0.0200076 | 6.80E-06 | 0.327448 | FALSE | 20.24995502 |
| 56 | rs78037977 | -0.129995 | 0.0289988 | 7.37E-06 | 0.499338 | FALSE | 20.09524212 |
| 57 | rs7805116 | -0.112945 | 0.0249878 | 6.18E-06 | 0.739319 | FALSE | 20.4304521 |
| 58 | rs7839768 | -0.113953 | 0.0229905 | 7.18E-07 | 0.343054 | FALSE | 24.56714524 |
| 59 | rs7988301 | 0.11002 | 0.0240043 | 4.58E-06 | 0.754305 | FALSE | 21.00705583 |
| 60 | rs8056814 | 0.27801 | 0.0310012 | 3.03E-19 | 0.312237 | FALSE | 80.41995498 |
| 61 | rs868093 | 0.0800119 | 0.0180027 | 8.81E-06 | 0.991271 | FALSE | 19.75303704 |
| 62 | rs911263 | 0.0949702 | 0.0209934 | 6.07E-06 | 0.95037 | FALSE | 20.46487723 |
| 63 | rs9585056 | -0.116004 | 0.0210007 | 3.32E-08 | 0.416424 | FALSE | 30.5125418 |

| **Confounding** | | |
| --- | --- | --- |
| 1 | rs1456988 | Graves' disease |
| 2 | rs1534422 | Autoimmune thyroid diseases (Graves disease or Hashimoto's thyroiditis) |
| 3 | rs2111485 | Hypothyroidism |
| 4 | rs3087243 | Hypothyroidism |
| 5 | rs3184504 | Hypothyroidism |
| 6 | rs72928038 | Autoimmune thyroid diseases (Graves disease or Hashimoto's thyroiditis) |

**Supplementary Table 8:** The instrumental variables used in MR analysis between exposure (Ulcerative colitis) and outcome (Autoimmune thyroiditis).

|  | **SNP** | **beta.exposure** | **se.exposure** | **pval.exposure** | **pval.outcome** | **palindromic** | **F** |
| --- | --- | --- | --- | --- | --- | --- | --- |
| 1 | rs10274130 | 0.2618 | 0.0584 | 7.46E-06 | 0.800396 | FALSE | 20.09618362 |
| 2 | rs10737481 | 0.1682 | 0.0194 | 4.96E-18 | 0.494054 | FALSE | 75.17068764 |
| 3 | rs10748781 | -0.1237 | 0.0199 | 4.99E-10 | 0.0884647 | FALSE | 38.63965556 |
| 4 | rs10799837 | -0.1058 | 0.0197 | 7.25E-08 | 0.290196 | FALSE | 28.84289727 |
| 5 | rs111843429 | 0.4666 | 0.1031 | 5.99E-06 | 0.314675 | FALSE | 20.48198946 |
| 6 | rs115400937 | 0.4697 | 0.1048 | 7.39E-06 | 0.142968 | FALSE | 20.08716075 |
| 7 | rs118016560 | 0.2228 | 0.0452 | 8.27E-07 | 0.51858 | FALSE | 24.29704754 |
| 8 | rs12461480 | 0.1812 | 0.0359 | 4.40E-07 | 0.235931 | FALSE | 25.47577998 |
| 9 | rs12574688 | 0.1385 | 0.0279 | 6.74E-07 | 0.971905 | FALSE | 24.64286173 |
| 10 | rs12638810 | 0.0874 | 0.0197 | 9.70E-06 | 0.291744 | TRUE | 19.68296014 |
| 11 | rs1268339 | 0.1057 | 0.0228 | 3.66E-06 | 0.485399 | FALSE | 21.49217067 |
| 12 | rs12720356 | 0.1604 | 0.0342 | 2.76E-06 | 0.946605 | FALSE | 21.99664854 |
| 13 | rs1359946 | 0.1178 | 0.0252 | 2.82E-06 | 0.830234 | FALSE | 21.85191484 |
| 14 | rs138523818 | 0.2571 | 0.053 | 1.21E-06 | 0.142047 | FALSE | 23.53165183 |
| 15 | rs144120543 | 0.2845 | 0.0614 | 3.59E-06 | 0.742843 | FALSE | 21.46979013 |
| 16 | rs144929017 | 0.3325 | 0.0748 | 8.66E-06 | 0.646074 | FALSE | 19.75968894 |
| 17 | rs145568234 | 1.1793 | 0.0955 | 4.78E-35 | 0.808741 | FALSE | 152.4901719 |
| 18 | rs149452325 | 0.1702 | 0.0373 | 5.21E-06 | 0.458027 | FALSE | 20.82099347 |
| 19 | rs151180560 | 0.3009 | 0.0655 | 4.36E-06 | 0.561202 | TRUE | 21.10385409 |
| 20 | rs1556478 | -0.1 | 0.0208 | 1.55E-06 | 0.823657 | FALSE | 23.11390533 |
| 21 | rs16939895 | 0.1206 | 0.0251 | 1.49E-06 | 0.947315 | FALSE | 23.08591927 |
| 22 | rs16940202 | 0.1322 | 0.0263 | 5.12E-07 | 0.979534 | FALSE | 25.26686811 |
| 23 | rs1801274 | -0.16 | 0.0194 | 1.63E-16 | 0.954091 | FALSE | 68.01998087 |
| 24 | rs181316459 | 0.3413 | 0.0585 | 5.49E-09 | 0.00307206 | TRUE | 34.03775002 |
| 25 | rs183569498 | 0.3007 | 0.066 | 5.28E-06 | 0.375524 | FALSE | 20.75768825 |
| 26 | rs185897821 | 0.3623 | 0.0799 | 5.70E-06 | 0.412908 | FALSE | 20.5609468 |
| 27 | rs2205532 | -0.091 | 0.0193 | 2.37E-06 | 0.565564 | FALSE | 22.2314693 |
| 28 | rs2278300 | 0.1252 | 0.0205 | 9.93E-10 | 0.950996 | FALSE | 37.29932183 |
| 29 | rs2888323 | -0.1038 | 0.0217 | 1.69E-06 | 0.660919 | FALSE | 22.88101255 |
| 30 | rs2918392 | 0.1169 | 0.0206 | 1.35E-08 | 0.51033 | FALSE | 32.2028702 |
| 31 | rs297202 | -0.094 | 0.0213 | 9.84E-06 | 0.794578 | TRUE | 19.47585356 |
| 32 | rs3024493 | 0.1847 | 0.0273 | 1.26E-11 | 0.00462424 | FALSE | 45.77290719 |
| 33 | rs311832 | 0.1534 | 0.0344 | 8.34E-06 | 0.852324 | FALSE | 19.88537723 |
| 34 | rs34236350 | 0.1485 | 0.0232 | 1.52E-10 | 0.550392 | FALSE | 40.97103523 |
| 35 | rs36051895 | 0.1031 | 0.0208 | 7.21E-07 | 0.433228 | FALSE | 24.56917992 |
| 36 | rs3757387 | 0.0979 | 0.0197 | 6.57E-07 | 0.163786 | FALSE | 24.69635909 |
| 37 | rs3828058 | 0.1216 | 0.0204 | 2.65E-09 | 0.211576 | FALSE | 35.53094963 |
| 38 | rs3852206 | 0.0924 | 0.0209 | 9.78E-06 | 0.184045 | FALSE | 19.54570637 |
| 39 | rs4065985 | -0.1092 | 0.0193 | 1.46E-08 | 0.0302894 | TRUE | 32.01331579 |
| 40 | rs4266763 | 0.0952 | 0.0194 | 9.75E-07 | 0.0990878 | FALSE | 24.08077373 |
| 41 | rs4625 | 0.1065 | 0.0212 | 4.94E-07 | 0.560593 | FALSE | 25.2364053 |
| 42 | rs4817983 | -0.1707 | 0.0227 | 5.59E-14 | 0.271778 | TRUE | 56.54774981 |
| 43 | rs4839549 | 0.1608 | 0.0362 | 8.70E-06 | 0.871557 | FALSE | 19.73126583 |
| 44 | rs4921492 | 0.1054 | 0.0203 | 1.97E-07 | 0.736663 | FALSE | 26.95809168 |
| 45 | rs495406 | -0.099 | 0.0198 | 5.96E-07 | 0.143455 | FALSE | 25 |
| 46 | rs6017342 | 0.1285 | 0.0197 | 6.63E-11 | 0.126823 | FALSE | 42.5474761 |
| 47 | rs60443775 | 0.1577 | 0.0345 | 4.73E-06 | 0.069528 | FALSE | 20.89417349 |
| 48 | rs61832589 | 0.1378 | 0.0306 | 6.90E-06 | 0.149262 | FALSE | 20.27942244 |
| 49 | rs6479830 | 0.1105 | 0.0217 | 3.64E-07 | 0.273868 | FALSE | 25.93015354 |
| 50 | rs6592792 | -0.1088 | 0.0237 | 4.36E-06 | 0.371048 | FALSE | 21.07468532 |
| 51 | rs6658093 | 0.0932 | 0.0192 | 1.24E-06 | 0.879349 | FALSE | 23.56293403 |
| 52 | rs6960886 | -0.0901 | 0.0195 | 3.96E-06 | 0.068135 | FALSE | 21.34913872 |
| 53 | rs7134599 | 0.113 | 0.0205 | 3.82E-08 | 0.756157 | FALSE | 30.38429506 |
| 54 | rs72967865 | 0.4189 | 0.0925 | 5.95E-06 | 0.242376 | FALSE | 20.50865858 |
| 55 | rs7554511 | -0.1304 | 0.0225 | 6.41E-09 | 0.105635 | FALSE | 33.5884642 |
| 56 | rs7559180 | 0.1883 | 0.0397 | 2.12E-06 | 0.229738 | FALSE | 22.49674194 |
| 57 | rs76354758 | 0.2514 | 0.0558 | 6.59E-06 | 0.619136 | FALSE | 20.298416 |
| 58 | rs76866541 | 0.1618 | 0.0357 | 5.82E-06 | 0.761507 | FALSE | 20.54095364 |
| 59 | rs77662016 | 0.2249 | 0.0507 | 9.30E-06 | 0.390088 | FALSE | 19.67718606 |
| 60 | rs7936434 | 0.1157 | 0.0199 | 5.80E-09 | 0.0970465 | TRUE | 33.80341406 |
| 61 | rs7942108 | -0.1585 | 0.033 | 1.60E-06 | 0.0774141 | FALSE | 23.06910009 |
| 62 | rs79565325 | 0.1317 | 0.0296 | 8.51E-06 | 0.929036 | FALSE | 19.79648694 |
| 63 | rs79755370 | -0.3098 | 0.046 | 1.64E-11 | 0.48906 | FALSE | 45.35729679 |
| 64 | rs8003714 | 0.4522 | 0.0892 | 4.01E-07 | 0.877879 | FALSE | 25.69989845 |
| 65 | rs886114 | 0.0926 | 0.0204 | 5.82E-06 | 0.919778 | FALSE | 20.60447905 |
| 66 | rs889100 | -0.0899 | 0.0198 | 5.33E-06 | 0.455868 | TRUE | 20.61526885 |
| 67 | rs909333 | -0.109 | 0.0229 | 1.84E-06 | 0.546048 | FALSE | 22.65593715 |
| 68 | rs9260809 | -0.1107 | 0.0196 | 1.61E-08 | 0.153317 | FALSE | 31.89944294 |
| 69 | rs9268849 | -0.2107 | 0.022 | 8.12E-22 | 0.00634352 | FALSE | 91.72415289 |
| 70 | rs9886651 | 0.09 | 0.0194 | 3.45E-06 | 0.105746 | FALSE | 21.52194707 |
| 71 | rs990107 | -0.1203 | 0.0196 | 9.30E-10 | 0.829483 | FALSE | 37.67203769 |

**Supplementary Table 9:** The instrumental variables used in MR analysis between exposure (Crohn's disease) and outcome (Autoimmune thyroiditis).

|  | SNP | beta.exposure | se.exposure | pval.exposure | pval.outcome | palindromic | F |
| --- | --- | --- | --- | --- | --- | --- | --- |
| 1 | rs10096271 | -0.129404 | 0.0263 | 8.76E-07 | 0.313743 | FALSE | 24.20939325 |
| 2 | rs10164254 | 0.1207 | 0.0269 | 7.36E-06 | 0.69174 | FALSE | 20.13306892 |
| 3 | rs10183992 | -0.259496 | 0.0583 | 8.48E-06 | 0.445255 | FALSE | 19.81181327 |
| 4 | rs1056441 | 0.167 | 0.0255 | 5.44E-11 | 0.297402 | FALSE | 42.88965782 |
| 5 | rs10748781 | -0.219102 | 0.0238 | 3.72E-20 | 0.0884647 | FALSE | 84.74981711 |
| 6 | rs10761659 | 0.212006 | 0.0237 | 3.42E-19 | 0.797311 | FALSE | 80.02019626 |
| 7 | rs111284483 | -0.159899 | 0.0335 | 1.77E-06 | 0.842625 | FALSE | 22.78252635 |
| 8 | rs11209026 | -0.995199 | 0.0639 | 1.05E-54 | 0.390528 | FALSE | 242.5594201 |
| 9 | rs11236797 | 0.181104 | 0.0231 | 4.85E-15 | 0.0354169 | FALSE | 61.4656 |
| 10 | rs11265502 | -0.210099 | 0.0428 | 9.18E-07 | 0.352943 | FALSE | 24.09685879 |
| 11 | rs114607072 | 0.441797 | 0.0629 | 2.20E-12 | 0.651633 | FALSE | 49.33376197 |
| 12 | rs11564236 | 0.519093 | 0.0595 | 2.85E-18 | 0.461152 | TRUE | 76.11257472 |
| 13 | rs116475860 | 0.417092 | 0.0894 | 3.11E-06 | 0.976224 | TRUE | 21.76650407 |
| 14 | rs117392501 | 0.313898 | 0.0679 | 3.83E-06 | 0.885243 | FALSE | 21.37162517 |
| 15 | rs117903731 | 0.533497 | 0.1024 | 1.87E-07 | 0.0407183 | FALSE | 27.1433877 |
| 16 | rs11812796 | -0.107997 | 0.0239 | 6.32E-06 | 0.26099 | FALSE | 20.41867616 |
| 17 | rs12194825 | -0.171904 | 0.0298 | 8.00E-09 | 0.204202 | TRUE | 33.27663756 |
| 18 | rs12437718 | -0.118299 | 0.0267 | 9.56E-06 | 0.917558 | FALSE | 19.63087349 |
| 19 | rs1250573 | -0.170895 | 0.0264 | 9.01E-11 | 0.714404 | FALSE | 41.90355404 |
| 20 | rs1267493 | 0.132903 | 0.0287 | 3.81E-06 | 0.269726 | FALSE | 21.44399885 |
| 21 | rs12692254 | 0.301402 | 0.0232 | 1.86E-38 | 0.630922 | TRUE | 168.7781763 |
| 22 | rs12694846 | 0.128299 | 0.0259 | 7.48E-07 | 0.755435 | FALSE | 24.53844367 |
| 23 | rs12717899 | 0.159198 | 0.0289 | 3.59E-08 | 0.995182 | FALSE | 30.3444681 |
| 24 | rs12827915 | 0.110199 | 0.0232 | 1.99E-06 | 0.401701 | FALSE | 22.56209052 |
| 25 | rs1292045 | 0.107296 | 0.0231 | 3.45E-06 | 0.688456 | FALSE | 21.57461745 |
| 26 | rs1297271 | -0.154901 | 0.0237 | 6.28E-11 | 0.0660389 | FALSE | 42.71808257 |
| 27 | rs13135092 | 0.221495 | 0.0389 | 1.21E-08 | 0.428189 | FALSE | 32.4211676 |
| 28 | rs13140783 | 0.202704 | 0.0438 | 3.60E-06 | 0.0943127 | FALSE | 21.41787682 |
| 29 | rs1321859 | -0.117602 | 0.0248 | 2.14E-06 | 0.000115027 | FALSE | 22.48671697 |
| 30 | rs13389477 | -0.131898 | 0.0285 | 3.71E-06 | 0.87398 | FALSE | 21.418384 |
| 31 | rs138350464 | -0.372906 | 0.0821 | 5.56E-06 | 0.258435 | FALSE | 20.63062704 |
| 32 | rs138953185 | -0.406406 | 0.0803 | 4.17E-07 | 0.0538233 | FALSE | 25.61469161 |
| 33 | rs140054334 | 0.349797 | 0.0628 | 2.57E-08 | 0.382172 | FALSE | 31.02507739 |
| 34 | rs142298794 | 0.482698 | 0.1054 | 4.65E-06 | 0.0856545 | FALSE | 20.97344527 |
| 35 | rs1456896 | 0.139301 | 0.0251 | 2.90E-08 | 0.661704 | FALSE | 30.8007311 |
| 36 | rs147018773 | 0.321699 | 0.0375 | 8.89E-18 | 0.309325 | FALSE | 73.59306425 |
| 37 | rs147684209 | 0.154901 | 0.0244 | 2.34E-10 | 0.943699 | FALSE | 40.30220337 |
| 38 | rs147799373 | 0.237496 | 0.051 | 3.18E-06 | 0.165187 | FALSE | 21.68564014 |
| 39 | rs148844907 | 0.958001 | 0.1419 | 1.47E-11 | 0.330255 | TRUE | 45.57924572 |
| 40 | rs151314883 | -0.223994 | 0.0327 | 7.12E-12 | 0.200187 | FALSE | 46.92208104 |
| 41 | rs1541953 | -0.114703 | 0.0228 | 5.03E-07 | 0.340174 | TRUE | 25.30928403 |
| 42 | rs1624017 | -0.145597 | 0.0284 | 3.06E-07 | 0.966578 | FALSE | 26.28259077 |
| 43 | rs168053 | -0.110005 | 0.0242 | 5.71E-06 | 0.866961 | FALSE | 20.66303535 |
| 44 | rs16903081 | -0.169599 | 0.036 | 2.49E-06 | 0.704403 | FALSE | 22.19430617 |
| 45 | rs16949819 | 0.139699 | 0.0266 | 1.46E-07 | 0.0126238 | FALSE | 27.5818455 |
| 46 | rs17156189 | -0.4524 | 0.0977 | 3.67E-06 | 0.142949 | FALSE | 21.44154447 |
| 47 | rs1721229 | 0.715099 | 0.1533 | 3.07E-06 | 0.158767 | FALSE | 21.75945591 |
| 48 | rs174567 | 0.119302 | 0.024 | 6.77E-07 | 0.173583 | FALSE | 24.71001251 |
| 49 | rs17643535 | 0.478499 | 0.0936 | 3.14E-07 | 0.506924 | TRUE | 26.13426987 |
| 50 | rs1775452 | 0.125204 | 0.0244 | 2.78E-07 | 0.551044 | TRUE | 26.33035746 |
| 51 | rs183183311 | 0.510101 | 0.1058 | 1.42E-06 | 0.417442 | FALSE | 23.2456136 |
| 52 | rs1873625 | 0.180704 | 0.0243 | 1.09E-13 | 0.640078 | FALSE | 55.29972669 |
| 53 | rs1887428 | -0.168099 | 0.0243 | 4.22E-12 | 0.244958 | TRUE | 47.8539413 |
| 54 | rs1932990 | 0.152901 | 0.0263 | 6.02E-09 | 0.0168085 | FALSE | 33.79941274 |
| 55 | rs1961812 | -0.187499 | 0.0409 | 4.68E-06 | 0.411128 | FALSE | 21.0160598 |
| 56 | rs2021511 | -0.123604 | 0.0264 | 2.75E-06 | 0.994962 | FALSE | 21.92084024 |
| 57 | rs2076756 | 0.399806 | 0.0242 | 3.24E-61 | 0.616453 | FALSE | 272.9404372 |
| 58 | rs212388 | -0.117298 | 0.0236 | 6.36E-07 | 0.00764522 | FALSE | 24.70342718 |
| 59 | rs2129944 | -0.1562 | 0.0271 | 7.81E-09 | 0.821063 | FALSE | 33.22182432 |
| 60 | rs2181783 | -0.114703 | 0.023 | 6.31E-07 | 0.034408 | FALSE | 24.87103631 |
| 61 | rs2188962 | 0.212398 | 0.0228 | 1.36E-20 | 0.36256 | FALSE | 86.78229918 |
| 62 | rs2476601 | 0.219102 | 0.0425 | 2.50E-07 | 9.17E-13 | FALSE | 26.57754265 |
| 63 | rs2492795 | -0.1313 | 0.0291 | 6.31E-06 | 0.333293 | FALSE | 20.35839208 |
| 64 | rs2505640 | -0.145701 | 0.0237 | 7.61E-10 | 0.339556 | FALSE | 37.79447988 |
| 65 | rs2581828 | -0.1095 | 0.0232 | 2.40E-06 | 0.606273 | TRUE | 22.27677244 |
| 66 | rs2675670 | 0.1074 | 0.0232 | 3.79E-06 | 0.263517 | TRUE | 21.43051427 |
| 67 | rs2712894 | -0.121704 | 0.0264 | 4.07E-06 | 0.341683 | FALSE | 21.2521 |
| 68 | rs281379 | 0.139797 | 0.0238 | 4.26E-09 | 0.308722 | FALSE | 34.50180285 |
| 69 | rs28701841 | 0.224303 | 0.0373 | 1.85E-09 | 0.595568 | FALSE | 36.16200491 |
| 70 | rs2923396 | -0.1112 | 0.0229 | 1.13E-06 | 0.343378 | FALSE | 23.57971816 |
| 71 | rs2968898 | -0.151195 | 0.0336 | 6.94E-06 | 0.367899 | FALSE | 20.24866074 |
| 72 | rs3024505 | 0.177903 | 0.0302 | 3.90E-09 | 0.00448291 | FALSE | 34.70185234 |
| 73 | rs3091315 | -0.179501 | 0.0263 | 9.52E-12 | 0.393815 | FALSE | 46.58244156 |
| 74 | rs34620647 | -0.177703 | 0.0356 | 6.06E-07 | 0.871718 | FALSE | 24.91664263 |
| 75 | rs355741 | 0.106595 | 0.023 | 3.43E-06 | 0.794043 | FALSE | 21.47919475 |
| 76 | rs3810936 | 0.207799 | 0.0263 | 2.46E-15 | 0.741208 | FALSE | 62.42742327 |
| 77 | rs3933040 | 0.364606 | 0.0806 | 6.12E-06 | 0.264756 | TRUE | 20.463388 |
| 78 | rs4077515 | 0.215901 | 0.0235 | 4.37E-20 | 0.1061 | FALSE | 84.40605125 |
| 79 | rs439409 | -0.141404 | 0.0268 | 1.35E-07 | 0.595312 | FALSE | 27.83901094 |
| 80 | rs444210 | 0.163402 | 0.0229 | 1.02E-12 | 0.312809 | FALSE | 50.91476822 |
| 81 | rs4653443 | 0.132501 | 0.0297 | 7.96E-06 | 0.314456 | FALSE | 19.90331486 |
| 82 | rs4817987 | -0.128402 | 0.0272 | 2.32E-06 | 0.27422 | FALSE | 22.2846475 |
| 83 | rs4820091 | 0.171702 | 0.0282 | 1.22E-09 | 0.227331 | FALSE | 37.07255269 |
| 84 | rs4851586 | -0.168899 | 0.0261 | 9.94E-11 | 0.956083 | FALSE | 41.87676664 |
| 85 | rs4902642 | -0.129198 | 0.0236 | 4.34E-08 | 0.738542 | FALSE | 29.97005746 |
| 86 | rs510372 | -0.109804 | 0.024 | 4.58E-06 | 0.318479 | FALSE | 20.93215003 |
| 87 | rs55690317 | 0.183995 | 0.0391 | 2.50E-06 | 0.318073 | FALSE | 22.14412519 |
| 88 | rs55897627 | 0.302901 | 0.0647 | 2.84E-06 | 0.535934 | FALSE | 21.91759274 |
| 89 | rs56062135 | 0.193097 | 0.0269 | 7.45E-13 | 0.076745 | FALSE | 51.52838049 |
| 90 | rs599077 | -0.127003 | 0.0268 | 2.12E-06 | 0.790022 | FALSE | 22.45734296 |
| 91 | rs62123096 | -0.138699 | 0.0311 | 7.94E-06 | 0.153454 | FALSE | 19.88959233 |
| 92 | rs62324212 | 0.1058 | 0.0238 | 8.88E-06 | 0.855862 | FALSE | 19.76138691 |
| 93 | rs6588052 | -0.1381 | 0.0256 | 6.94E-08 | 0.266259 | TRUE | 29.10096741 |
| 94 | rs6588243 | 0.131704 | 0.0234 | 1.78E-08 | 0.705135 | FALSE | 31.67861717 |
| 95 | rs6704109 | 0.202002 | 0.0256 | 2.77E-15 | 0.725422 | FALSE | 62.26319581 |
| 96 | rs6762648 | -0.136198 | 0.0285 | 1.74E-06 | 0.68548 | TRUE | 22.83766723 |
| 97 | rs6810216 | -0.104198 | 0.0236 | 9.64E-06 | 0.212143 | FALSE | 19.49372164 |
| 98 | rs6830046 | 0.516207 | 0.1043 | 7.52E-07 | 0.264033 | FALSE | 24.49509692 |
| 99 | rs6873866 | -0.168096 | 0.0239 | 2.06E-12 | 0.61772 | FALSE | 49.4673854 |
| 100 | rs697693 | 0.172296 | 0.0281 | 8.36E-10 | 0.219634 | FALSE | 37.5956632 |
| 101 | rs7237362 | 0.107196 | 0.0239 | 7.44E-06 | 0.0165421 | FALSE | 20.11691395 |
| 102 | rs72727394 | 0.127196 | 0.0281 | 5.84E-06 | 0.83511 | FALSE | 20.48963718 |
| 103 | rs72798422 | 0.590392 | 0.0508 | 3.19E-31 | 0.524274 | FALSE | 135.0683217 |
| 104 | rs744166 | -0.129299 | 0.0233 | 2.92E-08 | 0.95505 | FALSE | 30.79487815 |
| 105 | rs75140818 | 0.280302 | 0.0609 | 4.23E-06 | 0.561128 | FALSE | 21.18447998 |
| 106 | rs7522462 | -0.122405 | 0.0262 | 3.10E-06 | 0.122573 | FALSE | 21.82708471 |
| 107 | rs754209 | 0.125901 | 0.0245 | 2.65E-07 | 0.991693 | FALSE | 26.40743324 |
| 108 | rs7713270 | 0.296602 | 0.0241 | 6.97E-35 | 0.866362 | FALSE | 151.4656194 |
| 109 | rs7714401 | 0.159403 | 0.0244 | 6.20E-11 | 0.834908 | TRUE | 42.67891093 |
| 110 | rs78487399 | 0.2259 | 0.037 | 1.03E-09 | 0.241149 | TRUE | 37.27597516 |
| 111 | rs80262450 | 0.283102 | 0.0353 | 1.08E-15 | 0.989064 | FALSE | 64.31858245 |
| 112 | rs8092079 | -0.247102 | 0.0546 | 5.94E-06 | 0.440794 | FALSE | 20.48175824 |
| 113 | rs8178977 | 0.1928 | 0.0274 | 2.06E-12 | 0.326868 | TRUE | 49.51228089 |
| 114 | rs907092 | 0.130396 | 0.0228 | 1.01E-08 | 0.0619955 | FALSE | 32.70836568 |
| 115 | rs921720 | 0.162895 | 0.0237 | 6.40E-12 | 0.0967231 | FALSE | 47.24097104 |
| 116 | rs9491697 | 0.107296 | 0.0231 | 3.55E-06 | 0.892382 | FALSE | 21.57461745 |
| 117 | rs9493888 | 0.137804 | 0.031 | 8.87E-06 | 0.999205 | FALSE | 19.76060605 |
| 118 | rs9573018 | 0.108098 | 0.0238 | 5.82E-06 | 0.0236701 | TRUE | 20.62915332 |
| 119 | rs9675039 | -0.123796 | 0.0273 | 5.68E-06 | 0.790031 | FALSE | 20.56306889 |
| 120 | rs988783 | -0.351403 | 0.0741 | 2.12E-06 | 0.258261 | FALSE | 22.48922625 |
| 121 | rs9934775 | -0.159101 | 0.0318 | 5.46E-07 | 0.294327 | FALSE | 25.03177109 |

**Supplementary Table 10:** MR analysis process of exposures and outcomes.

| **Exposure** | **Outcome** | **Heterogeneity** | | **MR-egger** | | **MR-PRESSO Global** | |
| --- | --- | --- | --- | --- | --- | --- | --- |
|  |  | **Cochran’s Q** | **P value** | **Egger intercept** | **P value** | **Rssobs** | **P value** |
| RA | AIT | 79.32 | 0.080 | 3.56E-05 | 0.999 | 81.66 | 0.060 |
| T1D | AIT | 67.86 | 0.114 | 0.049 | 0.018 | 74.11 | 0.100 |
| CD | AIT | 123.45 | 0.056 | -0.030 | 0.156 | 125.68 | 0.073 |
| UC | AIT | 81.22 | 0.114 | -0.039 | 0.217 | 93.5 | 0.051 |
| SS | AIT | 13.92 | 0.306 | 0.013 | 0.885 | 16.78 | 0.300 |
| SLE | AIT | 22.66 | 0.252 | 0.004 | 0.918 | 27.16 | 0.245 |
| MS | AIT | 82.71 | 0.015 | -0.034 | 0.140 | 32.92 | 0.832 |
| AS | AIT | 37.00 | 0.561 | -0.021 | 0.280 | 39.32 | 0.552 |

MR: mendelian randomization; IVW: inverse variance weighted; Autoimmune thyroiditis (AIT); rheumatoid arthritis (RA) ; Type 1 diabetes(T1D); systemic lupus erythematosus (SLE), Sjögren’s syndrome (SS), Ankylosing Spondylitis (AS), Multiple sclerosis (MS), Crohn’s disease (CD); ulcerative colitis (UC)

**Supplementary Table 11**: MR results of causal relationships between gut microbiota and pulmonary embolism.

| Exposure | Outcome | Methods | OR(95%CI) | P value |
| --- | --- | --- | --- | --- |
| RA | AIT | MR Egger | 1.42(0.80,2.53) | 0.238 |
| RA | AIT | Weighted median | 1.29(0.91,1.84) | 0.152 |
| RA | AIT | Simple mode | 1.06(0.49,2.31) | 0.883 |
| RA | AIT | Weighted mode | 1.31(0.75,2.27) | 0.346 |
| T1D | AIT | MR Egger | 0.99(0.77,1.26) | 0.918 |
| T1D | AIT | Weighted median | 1.04(0.86,1.27) | 0.676 |
| T1D | AIT | Simple mode | 1.21(0.81,1.80) | 0.363 |
| T1D | AIT | Weighted mode | 1.08(0.89,1.31) | 0.430 |
| CD | AIT | MR Egger | 1.25(0.99,1.57) | 0.063 |
| CD | AIT | Weighted median | 1.11(0.96,1.28) | 0.156 |
| CD | AIT | Simple mode | 1.04(0.77,1.40) | 0.817 |
| CD | AIT | Weighted mode | 1.07(0.87,1.31) | 0.511 |
| UC | AIT | MR Egger | 0.70(0.45,1.10) | 0.128 |
| UC | AIT | Weighted median | 0.92(0.73,1.16) | 0.467 |
| UC | AIT | Simple mode | 1.00(0.53,1.89) | 0.989 |
| UC | AIT | Weighted mode | 0.90(0.53,1.53) | 0.705 |
| SS | AIT | MR Egger | 0.97(0.67,1.40) | 0.868 |
| SS | AIT | Weighted median | 0.88(0.76,1.02) | 0.093 |
| SS | AIT | Simple mode | 0.85(0.65,1.11) | 0.263 |
| SS | AIT | Weighted mode | 0.85(0.67,1.08) | 0.221 |
| SLE | AIT | MR Egger | 1.13(0.98,1.32) | 0.108 |
| SLE | AIT | Weighted median | 1.05(0.90,1.21) | 0.558 |
| SLE | AIT | Simple mode | 0.97(0.72.1.30) | 0.816 |
| SLE | AIT | Weighted mode | 1.02(0.87,1.20) | 0.796 |
| MS | AIT | MR Egger | 1.11(0.92,1.34) | 0.278 |
| MS | AIT | Weighted median | 1.10(0.95,1.27) | 0.219 |
| MS | AIT | Simple mode | 1.03(0.73,1.47) | 0.858 |
| MS | AIT | Weighted mode | 1.09(0.92,1.28) | 0.331 |
| AS | AIT | MR Egger | 0.52(0.22,1.24) | 0.150 |
| AS | AIT | Weighted median | 0.96(0.46,2.00) | 0.913 |
| AS | AIT | Simple mode | 0.82(0.14,4.99) | 0.833 |
| AS | AIT | Weighted mode | 0.74(0.35,1.58) | 0.444 |

Autoimmune thyroiditis (AIT); rheumatoid arthritis (RA) ; Type 1 diabetes(T1D); systemic lupus erythematosus (SLE), Sjögren’s syndrome (SS), Ankylosing Spondylitis (AS), Multiple sclerosis (MS), Crohn’s disease (CD); ulcerative colitis (UC)
